# ESA1 regulates meiotic chromosome axis and crossover frequency via acetylating histone H4

**DOI:** 10.1093/nar/gkab722

**Published:** 2021-08-20

**Authors:** Ying Wang, Binyuan Zhai, Taicong Tan, Xiao Yang, Jiaming Zhang, Meihui Song, Yingjin Tan, Xuan Yang, Tingting Chu, Shuxian Zhang, Shunxin Wang, Liangran Zhang

**Affiliations:** Center for Reproductive Medicine, Cheeloo College of Medicine, State Key Laboratory of Microbial Technology, Shandong University, China; Center for Reproductive Medicine, Cheeloo College of Medicine, State Key Laboratory of Microbial Technology, Shandong University, China; Center for Reproductive Medicine, Cheeloo College of Medicine, State Key Laboratory of Microbial Technology, Shandong University, China; Center for Reproductive Medicine, Cheeloo College of Medicine, State Key Laboratory of Microbial Technology, Shandong University, China; Center for Reproductive Medicine, Cheeloo College of Medicine, State Key Laboratory of Microbial Technology, Shandong University, China; Center for Reproductive Medicine, Cheeloo College of Medicine, State Key Laboratory of Microbial Technology, Shandong University, China; Center for Reproductive Medicine, Cheeloo College of Medicine, State Key Laboratory of Microbial Technology, Shandong University, China; Center for Reproductive Medicine, Cheeloo College of Medicine, State Key Laboratory of Microbial Technology, Shandong University, China; Center for Reproductive Medicine, Cheeloo College of Medicine, State Key Laboratory of Microbial Technology, Shandong University, China; Center for Reproductive Medicine, Cheeloo College of Medicine, State Key Laboratory of Microbial Technology, Shandong University, China; Center for Reproductive Medicine, Cheeloo College of Medicine, State Key Laboratory of Microbial Technology, Shandong University, China; National Research Center for Assisted Reproductive Technology and Reproductive Genetics, Shandong University, Jinan, Shandong 250012, China; Key Laboratory of Reproductive Endocrinology of Ministry of Education, Jinan, Shandong250001, China; Shandong Provincial Clinical Research Center for Reproductive Health, Jinan, Shandong 250012, China; Center for Reproductive Medicine, Cheeloo College of Medicine, State Key Laboratory of Microbial Technology, Shandong University, China; Advanced Medical Research Institute, Shandong University, Jinan, Shandong250012, China; Shandong Provincial Key Laboratory of Animal Resistance Biology, College of Life Sciences, Shandong Normal University, Jinan250014, Shandong, China

## Abstract

Meiotic recombination is integrated into and regulated by meiotic chromosomes, which is organized as loop/axis architecture. However, the regulation of chromosome organization is poorly understood. Here, we show Esa1, the NuA4 complex catalytic subunit, is constitutively expressed and localizes on chromatin loops during meiosis. Esa1 plays multiple roles including homolog synapsis, sporulation efficiency, spore viability, and chromosome segregation in meiosis. Detailed analyses show the meiosis-specific depletion of Esa1 results in decreased chromosome axis length independent of another axis length regulator Pds5, which further leads to a decreased number of Mer2 foci, and consequently a decreased number of DNA double-strand breaks, recombination intermediates, and crossover frequency. However, Esa1 depletion does not impair the occurrence of the obligatory crossover required for faithful chromosome segregation, or the strength of crossover interference. Further investigations demonstrate Esa1 regulates chromosome axis length via acetylating the N-terminal tail of histone H4 but not altering transcription program. Therefore, we firstly show a non-chromosome axis component, Esa1, acetylates histone H4 on chromatin loops to regulate chromosome axis length and consequently recombination frequency but does not affect the basic meiotic recombination process. Additionally, Esa1 depletion downregulates middle induced meiotic genes, which probably causing defects in sporulation and chromosome segregation.

## INTRODUCTION

Meiosis is essential to produce gametes with half chromosome complements of their parents. This process includes a single round of DNA replication followed by segregation of homologous chromosomes (homologs) during meiosis I (MI) and then segregation of sister chromatids during meiosis II (MII).

Meiotic recombination is required for proper homologous chromosome segregation in most organisms and also plays a central role in evolutionary adaption via promoting genetic diversity. At DNA molecular level, meiotic recombination is initiated by the evolutionarily conserved Spo11 transesterase mediated DNA double-strand breaks (DSBs), which are primarily repaired with homologous templates as crossovers (COs) or non-crossovers (NCOs) (1–3).

Recombination occurs in the context of chromosomes and is regulated both globally and locally by chromosome architecture from DSBs formation to mature recombination products (4–11). (i) Neither DSBs nor COs are randomly distributed on chromosomes and they occur at preferred ‘hot’ spots. However, their localization also shows a great variability between nuclei (1,12–15). (ii) DSBs at different positions on chromosomes have different probabilities to be repaired with its homologous templates and have different probabilities to be repaired as COs (8,13,16–21). Therefore, DSB hot spots are not always CO hot spots. (iii) Each pair of homolog obtains at least one CO (the obligatory CO), and CO interference forces multiple COs along a pair of homologs stay further away from each other than would be expected by chance (12,22,23). (4) CO homeostasis keeps CO number relatively constant despite variations in DSB numbers in the natural population or under altered condition (12,22,24–26).

The meiotic chromosome is organized as a linear array of loops, the base of which is the axis composed of a protein/DNA meshwork (27). Along meiotic pachytene chromosomes, the number of loops per micron of chromosome axis is evolutionarily conserved (∼20) among different organisms although they have very different genome sizes (27). Therefore, axis length is determined by and negatively correlated with loop size (28–32). For example, in many species including human, among the two sexes, the one has longer or shorter axis is accompanied by shorter or longer loops (28,33).

Current results support that chromosome axis length regulates the number of meiotic DSBs and homologous DNA recombination frequency (28,32,34). For example, compared with human males, human females have longer meiotic chromosome axis and consequently more DSBs and COs (28,33). However, what determines the chromosome loop/axis organization is unknown.

In both mitosis and meiosis, chromosome architecture is modulated by histone modifications including methylation, phosphorylation, ubiquitylation, and acetylation, etc. These modifications may affect interactions between histones, between histone and DNA, and between chromatin and non-histones to influence higher-order chromatin structure both globally and locally (35,36). Among different types of histone modifications, acetylation neutralizes the basic charge of the lysine to relax chromatin (36). Histone acetylation plays important roles in many important processes such as transcription and DNA repair in mitosis (37–39), and meiotic DSB formation, CO recombination and the dynamics of the synaptonemal complex (SC) in meiosis (9,40–44).

The NuA4 complex is mainly responsible for histone H4 acetylation, the catalytic subunit of which is Esa1, the only essential acetyltransferase in yeast (45–47). Esa1 and its human ortholog Tip60 belong to the MYST family of acetyltransferases and play critical roles in transcription, DNA replication, and DNA damage repair (48–52). Therefore, it is of great interest to explore the roles of Esa1 in meiosis, especially the possible roles in chromosome organization and meiotic recombination.

Here, we investigated the roles of Esa1 during meiosis. We showed that Esa1 is constitutively expressed and localizes on chromatin loops during meiosis. The meiosis-specific depletion of Esa1 results in defects in homolog synapsis, sporulation efficiency, spore viability, and chromosome segregation. Esa1 depletion decreases meiotic chromosome axis length independent of another axis regulator Pds5, which results in a reduced number of Mer2 foci required for efficient DSB formation, and as a result, decreased frequencies of DSBs, recombination intermediates, and COs. However, Esa1 depletion does not affect the basic meiotic recombination process because it does not impair the occurrence of the obligatory CO or the strength of CO interference. Further investigations demonstrated that Esa1 regulates chromosome axis length via acetylating histone H4 N-terminal tail. These results suggest Esa1 has multiple roles during meiosis and an important role is to acetylate histone H4 on chromatin loops to regulates chromosome axis length and consequently recombination frequency.

## MATERIALS AND METHODS

### Plasmids

The plasmid pRS306-hhf1-N4-19Δ-URA3 was constructed by overlap extension PCR as below. The fragments containing HHF1 N1-3 and HHF1 N20-stop codon were amplified by PCR with genomic DNA as templates (primers ZP503F/ZP503R and ZP504F/ZP504R, respectively; see Supplementary Table S1 for primer sequence). The two PCR fragments were fused together by overlap extension PCR with the two fragments as templates (primers ZP503F and ZP504R). The resultant fragment containing *hhf1 N4-19Δ* was treated with SpeI and XhoI enzymes and cloned into pRS306 vector to generate plasmid pRS306-hhf1-N4-19Δ-URA3. The plasmid pRS306-hhf2-N4-19Δ-URA3 was similarly constructed. Primers ZP507F/ZP507R and ZP508F/ZP508R were used to get the two half fragments and primers ZP507F/ZP508R were used for overlap extension PCR.

The plasmid pRS306-hhf1-K5,8,12,16Q-URA3 was constructed by overlap extension PCR as described below. The hhf1-K5,8,12,16Q mutations were introduced by PCR with genomic DNA as templates and mutations on primers (ZP503F/ZP1003 and ZP1004/ZP504R, respectively). The two amplified fragments with mutations were fused together by overlap extension PCR (primers ZP503F/ZP504R). The resultant fragment containing hhf1-K5,8,12,16Q was cloned into pRS306 after treated with SpeI and XhoI enzymes to generate pRS306-hhf1-K5,8,12,16Q-URA3. Plasmids pRS306-hhf1-K5,8,12,16R-URA3, pRS306-hhf2-K5,8,12,16Q-URA3, and pRS306-hhf2-K5,8,12,16R-URA3 were similarly constructed. Primer pairs ZP503F/ZP1005, ZP1006/ZP504R, and ZP503F/ZP504R were used for pRS306-hhf1-K5,8,12,16R-URA3; ZP507F/ZP1007, ZP1008/ZP508R, and ZP507F/ZP508R were used for pRS306-hhf2-K5,8,12,16Q-URA3; ZP507F/ZP1009, ZP1010/ZP508R, and ZP507F/ZP508R were used for pRS306-hhf2-K5,8,12,16R-URA3.

### Yeast strains

The *Saccharomyces cerevisiae* strains used in this study are shown in Supplementary Table S2 and are of SK1 background, except for strains used for whole-genome re-sequencing, which were made by mating S96 and YJM789 haploids.

The *pCLB2-ESA1* strain was constructed by replacing its native promoter by the *CLB2* promoter using the polymerase chain reaction (PCR) method (53,54). The *ESA1-3HA* strain was constructed via tagging a triple-HA to the C-terminal of *ESA1* by the PCR method. The *MER2-3MYC* strain was constructed via inserting a triple-myc fragment between the 248th and 249th codons of *MER2* as previously described (55). These tagged proteins function normally in meiosis (Supplementary Figure S1). The *hhf1 N4-19Δ HHF2* and *HHF1 hhf2 N4-19Δ* strains were generated with AfeI treated pRS306-hhf1-N4-19Δ-URA3 and HindIII treated pRS306-hhf2-N4-19Δ-URA3, respectively, by two step gene replacement strategy (56). The opposite mating types of *hhf1 N4-19Δ HHF2* and *HHF1 hhf2 N4-19Δ* haploids were mated on YPD plates (1% yeast extract, 2% Bacto peptone, 2% glucose and 2% Bacto agar), the mixture was sporulated, tetrads were dissected onto YPD plates, and *hhf1 N4-19Δ hhf2 N4-19Δ* haploids were selected. MATa *hhf1 N4-19Δ hhf2 N4-19Δ* haploids show low mating efficiency. Therefore, the haploids with pHHF1-URA3 plasmids were mated with the *MATα hhf1 N4-19Δ hhf2 N4-19Δ* to get the diploids with pHHF1-URA3 plasmids, after that the pHHF1-URA3 plasmids were removed by plating cells onto 5-Fluoroorotic Acid (5-FOA) plates (0.1% 5-FOA, 0.2% uracil dropout mix, 0.02% uracil, 0.67% yeast nitrogen base without amino acids, 2% glucose and 2% Bacto agar). The *hhf K5,8,12,16Q* diploids and *hhf K5,8,12,16R* diploids were obtained using the similar methods. All mutants were confirmed by PCR in combination with DNA sequencing.

### Meiotic time course

The meiotic time course was performed as previously described (57). To get synchronous meiotic yeast cultures, cells were patched onto YPG plates (3% glycerol, 1% yeast extract, 2% Bacto peptone and 2% Bacto agar) incubated at 30°C overnight. Cells were streaked onto YPD plates (1% yeast extract, 2% Bacto peptone, 2% glucose, and 2% Bacto agar) and incubated at 30°C for two days. Healthy single colonies were inoculated into 4ml of YPD liquid medium and incubated at 30°C for 24 h with shaking. Appropriate amount of YPD liquid cultures was transferred into supplemented pre-sporulation liquid medium (SPS; 0.5% yeast extract, 1% Bacto peptone, 0.67% yeast nitrogen base without amino acids, 1% potassium acetate and 50 mM potassium biphthalate, pH 5.5) and cultured at 30°C for 16–18 h with shaking. Synchronized yeast cells were harvested and transferred to sporulation medium (SPM; 1% potassium acetate, 0.02% raffinose) to induce meiosis at 30°C. At each hour, samples were collected, fixed, and stained with 4,6-diamidino-2-phenylindole (DAPI) to determine meiotic divisions using a Zeiss fluorescence microscope (AxioImager.Z2) with an EMCCD camera.

### Sporulation efficiency and spore viability assays

Sporulation efficiency was determined by calculating the frequency of cells with asci visualized under a light microscopy. Tetrads were dissected onto YPD plates with a dissection microscope (SporePlay from Singer Instruments) and incubated at 30°C for 2–3 days, and then the viable spore patterns and spore viability were calculated.

### Cytological analysis

Samples were collected from synchronized cultures at appropriate time points for cytological analysis. To prepare chromosome spreads of yeast nuclei, cells were processed into spheroplasts with Zymolyase 100 T, spread on a clean slide with 1% Lipsol, and fixed by 3% paraformaldehyde containing 3.4% sucrose (24). For immunostaining, slides were dipped into 0.2% Photo-Flo for 30 s, transferred to Tris-Buffered Saline (TBS) (136 mM NaCl, 3 mM KCl and 25 mM Tris–Cl, pH 8.0), and incubated at room temperature for 15 min. Then the slides were dipped in 1% BSA for 10 min and incubated with appropriate primary (4°C overnight) and secondary (37°C for 2 h) antibodies. The primary antibodies used in this study: mouse monoclonal anti-Myc (Santa Cruz Biotechnology, Cat# sc-40), goat polyclonal anti-Zip1 (Santa Cruz Biotechnology, Cat# sc-48716), rabbit polyclonal anti-GFP (Thermo Fisher Scientific, Cat# A11122), rat monoclonal anti-HA (Roche, Cat# 11867423001), rabbit polyclonal anti-Rad51 (Santa Cruz Biotechnolog, Cat# sc-33626), rat polyclonal anti-Pds5 (prepared by Dia-An Biotech, Wuhan, China) and rabbit polyclonal anti-H4(K5ac + K8ac + K12ac + K16ac) (Abcam, Cat# ab10807). The following secondary antibodies were used in this study: Alexa 488-conjugated donkey anti-mouse/goat/rabbit (Thermo Fisher Scientific, Cat# A-21202/A-11055/A-21206), Alexa 555-conjugated donkey anti-rabbit (Thermo Fisher Scientific, Cat# A-31572), and Alexa 594-conjugated donkey anti-rat/goat (Thermo Fisher Scientific, Cat# A-21209/A-11058). Chromosomal DNA was stained with DAPI. Fluorescence images were acquired using a Zeiss fluorescence microscope (AxioImager.Z2) with an EMCCD camera. SC lengths and Zip3 focus positions were determined using ImageJ (https://imagej.nih.gov/ij) and transferred into an EXCEL worksheet for further analysis.

### Measurement of chromosome axis length

Chromosome axis length was determined as previously described (58). During pachytene, homologs are tightly connected from end to end by SC and axis length can be determined by measurement of Zip1 or Rec8 lines.

### Quantification of immunofluorescence intensity

Histone H4 acetylation level was estimated by their fluorescence intensity after immunostaining as previously described (58). To accurately compare fluorescence intensities of mutants with wild type, mutants and wild type were mixed and spread on the same slides. Rec8 in wild type strain was tagged with 3HA, allowing the mutants and wild type to be distinguished when immunostained with anti-HA antibodies. The same protocol was used for immunostaining, including buffers, the concentration of antibodies, incubation time, etc. After immunostaining, the same parameters were used in imaging. The fluorescence intensity was measured with ImageJ software. The background level was determined as below. For a nucleus, a chromosome fragment was randomly selected and a line perpendicular to it was drawn. The fluorescence intensity profile (signal intensity of each pixel) of this line was displaced as a normal distribution curve. The background pixel intensity was defined as the pixel intensity where the curve being flattened as horizontal lines. For each nucleus, three chromosome fragments were randomly selected and thus three background values were obtained. The average of these three values was defined as the background fluorescence intensity for each pixel in this nucleus. The target nucleus was circled. The number of pixels whose intensity above the average background was counted, and the sum of these pixel intensities is the total raw fluorescence intensity. The fluorescence intensity was obtained by raw fluorescence intensity subtracting the background fluorescence intensity. [Background intensity] = [background pixel intensity] × [the number of pixels whose intensity is above the average background].

### CO interference analysis

CO interference can be calculated by coefficient of coincidence (CoC) or gamma distribution analysis. For this purpose, SC length and CO-correlated marker Zip3 focus positions were measured on chromosome XV from the *lacO*/LacI-GFP marked end to the other end with ImageJ software. More than 200 nuclei were measured in each experiment for each strain. The data were recorded in an EXCEL worksheet for further analysis.

CoC analysis is the classic and robust way to measure crossover interference (12,59). For CoC analysis on chromosome XV, analyzed chromosomes were divided into 30 intervals with equal size (about 0.1 μm in length) as previously described (12). Each chromosome length was normalized to 100% and each Zip3 focus position was also normalized correspondingly. Each Zip3 focus was then assigned to a given interval according to its coordinate. The frequency of chromosomes bearing a Zip3 focus in each interval was calculated. For each pair of intervals, the “observed” frequency of double COs (Obs DCOs) is the frequency of chromosomes bearing Zip3 foci in both intervals, the “expected or predicted” frequency of double COs (Pred DCOs) is calculated as the product of the CO frequencies for the two individual intervals based on the hypothesis of independent occurrence, and the ratio of these two values is the CoC, i.e., CoC = (Obs DCO)/(Pred DCO). CoC values for all possible pairs of intervals can be easily calculated and each corresponding “inter-interval distance” is calculated as the distance between the midpoints of the two involved intervals. When CoC values from interval pairs with same inter-interval distances are averaged and plotted against corresponding inter-interval distance, a CoC curve can be obtained. Typically, the CoC value is close to zero or very small at short inter-interval distance, indicating strong CO interference. Along with increasing inter-interval distance, CoC values increase and finally fluctuate around one. This indicates reduced interference strength with increasing inter-interval distance and no interference at large distance. A CoC curve can be easily calculated by using an MATLAB application (https://app.box.com/s/hv91q2nrtq0cp9n8iy9m) as previous described (12).

The strength of CO interference can also be measured by the shape parameter of gamma distribution with a larger shape pramater indicating stronger interference. To estimate CO interference level by gamma distribution, the inter-adjacent Zip3 focus distances were calculated based on Zip3 focus data. The best-fit gamma distribution for inter-adjacent Zip3 focus distances from all chromosome XV were calculated by the maximum likelihood method using the ‘gamfit’ function in MATLAB (MathWorks).

### Chromosome compaction assay

Chromosome compaction was done according to previously described (57,60,61). The strain with *lacO/LacI-GFP* labelled both *TRP1* and *TEL4* on the same chromosome IV were used. Yeast cells were induced to synchronously enter meiosis and samples were collected at indicated time points. Chromosome spreads were prepared as described above and stained with both anti-Zip1 and anti-GFP antibodies. Chromosome compaction was determined by measuring the distances between the two GFP spots using ImageJ software.

### Western blot

Western blot was done according to previously described (24). Briefly, yeast cells were fixed and lysed in 20% trichloroacetic acid (TCA) using glass beads. The resultant pellet was extracted with Laemmli buffer and denatured in boiling water for 5 min, then the proteins were separated by SDS-PAGE and transferred to nitrocellulose filter membranes (Millipore, Cat# HATF00010). The mouse monoclonal anti-HA (Sigma, Cat# 11867423001), mouse monoclonal anti-PGK1 (Abcam, Cat# ab113687), rabbit polyclonal anti-H3K4me3 (active motif, Cat# 39159), rabbit polyclonal anti-Histone H4-C terminal (Affinity, Cat# DF6950), rabbit polyclonal anti-Acetyl-Histone H4(Lys8) (Affinity, Cat# AF4353), rabbit monoclonal anti-Acetyl-Histone H4(Lys12) (Cell Signaling Technology, Cat# 13944S), and rabbit polyclonal anti-Acetyl-Histone H4(Lys16) (Affinity, Cat# AF3636) antibodies were used for western blot. Quantification was performed using ImageJ software.

### Physical analysis

Chromosomal DNA preparation and physical analysis were carried out as previously described (62–64). Cell samples were collected at proper time points to extract genmic DNA. The extracted genomic DNA was digested with the XhoI enzyme for DSB and CO assay and digested with XhoI and NgoIV enzymes for CO/NCO assay. DNA was separated on a 1D gel by electrophoresis. DSBs and recombination products were detected via Southern hybridization using ^32^P-dCTP labeled ‘Probe A’ (65). Hybridizing signals were quantified by Quantity One software.

### Spore-specific fluorescence assay of chromosome mis-segregation

Spore-specific promoter driven YFP (*P^YKL050c^-YFP*) and RFP (*P^YKL050c^-RFP*) were integrated near the centromeres of the two homologous chromosome IX, respectively. Fresh cells were inoculated into 4ml of SPM liquid medium and incubated at 30°C for 2 days with shaking. Spore patterns with different fluorescence in tetrads were examined under a Zeiss fluorescence microscope (AxioImager.Z2). For the four spores in a tetrad with faithful segregated chromosomes, two spores express YFP and the other two express RFP. Tetrads with missegregated chromosome IX show aberrant patterns of yellow/red fluorescence. The frequency of chromosome IX mis-segregation was defined as the number of tetrads with mis-segregated chromosome IX divided by the number of total tetrads (tetrads without YFP/RFP spores were excluded in this analysis). At least two independent experiments were carried out. Totally 815 (WT) and 777 (*esa1-md*) tetrads were examined.

### Whole-genome re-sequencing

The haploid strain in S96 background and the haploid strain with the opposite mating type in YJM789 background were mated for 6 h on YPD plates and the mixture was sporulated in SPM at 30°C. Tetrads were dissected onto YPD plates. Only the tetrads with 4-viable spores were used for the following analysis. Each spore clone from a single tetrad was examined using allele-specific colony PCR to ensure all four clones are euploidies (66). DNA was prepared from each spore clone and sequenced at the company of Novogene using the Illumina HiSeq X Ten platform with 150 bp pair-end reads. The ReCombine program was used to determine the meiotic recombination events (67). The wild-type data include 10 tetrads from resequencing (58; SRA Bioproject Accession number PRJNA695084). The *esa1-md* resequencing data are available at NCBI (SRA Bioproject, accession number PRJNA747839).

### mRNA sequencing

Samples collected from synchronized cultures in SPM were flash frozen in liquid nitrogen for total RNA isolation. Total RNA was extracted and sequenced at the company of Novogene using Illumina HiSeq 4000 platform with 150 bp pair-end reads as previously described (68). Experiments were performed in biological triplicates. A small amount of reads containing adapter and low quality reads were removed from raw data. These clean reads were mapped to the reference genome (SacCer3) using Hisat2 v2.0.5 (69,70). mRNA abundance was determined using DESeq2 and differentially expressed genes between two samples were extracted when Benjamini–Hochberg adjusted *P* value < 0.05 and |log_2_(fold change)| ≥ 1 (71). Functional enrichment analysis of differentially expressed genes was performed using Metascape (http://metascape.org). Heatmap of the expression profiles for the differentially expressed genes were performed using OmicShare Tool (https://www.genedenovo.com). Gene expression data are available at NCBI (SRA Bioproject, accession number PRJNA748179).

### Quantitative reverse transcription PCR (RT-qPCR)

Total RNA were extracted with Spin Column Yeast Total RNA Purification Kit (Sangon Biotech, B518657-0050) using the manufacturer's protocol from the indicated samples. About 0.8 μg of total RNA was reverse transcribed using a HiScript™II Q RT SuperMix for qPCR (Vazyme, R223-01). The cDNA was diluted by 3 times and 1.5 μl cDNA was used for each reaction containing 2X Universal SYBR Green Fast qPCR Mix (ABclonal, RK21203) in a LightCycle 96 Real-Time PCR system (Roche). All reactions with specific forward and reverse primers were performed in triplicate. Primers were listed in Supplementary Table S3.

### Statistical analyses

The Student's *t-*test of means was used to calculate statistical significance in the average number of events between samples. Two proportion z-test was used to determine significance in the frequencies between samples. The levels of significance are indicated in figures: *P* ≥ 0.05 (n.s., not significant), *P* < 0.05 (*), *P* < 0.01 (**) and *P* < 0.001 (***).

## RESULTS

### Esa1 is constitutively expressed and localizes on chromatin loops during meiosis

**Figure 1.**
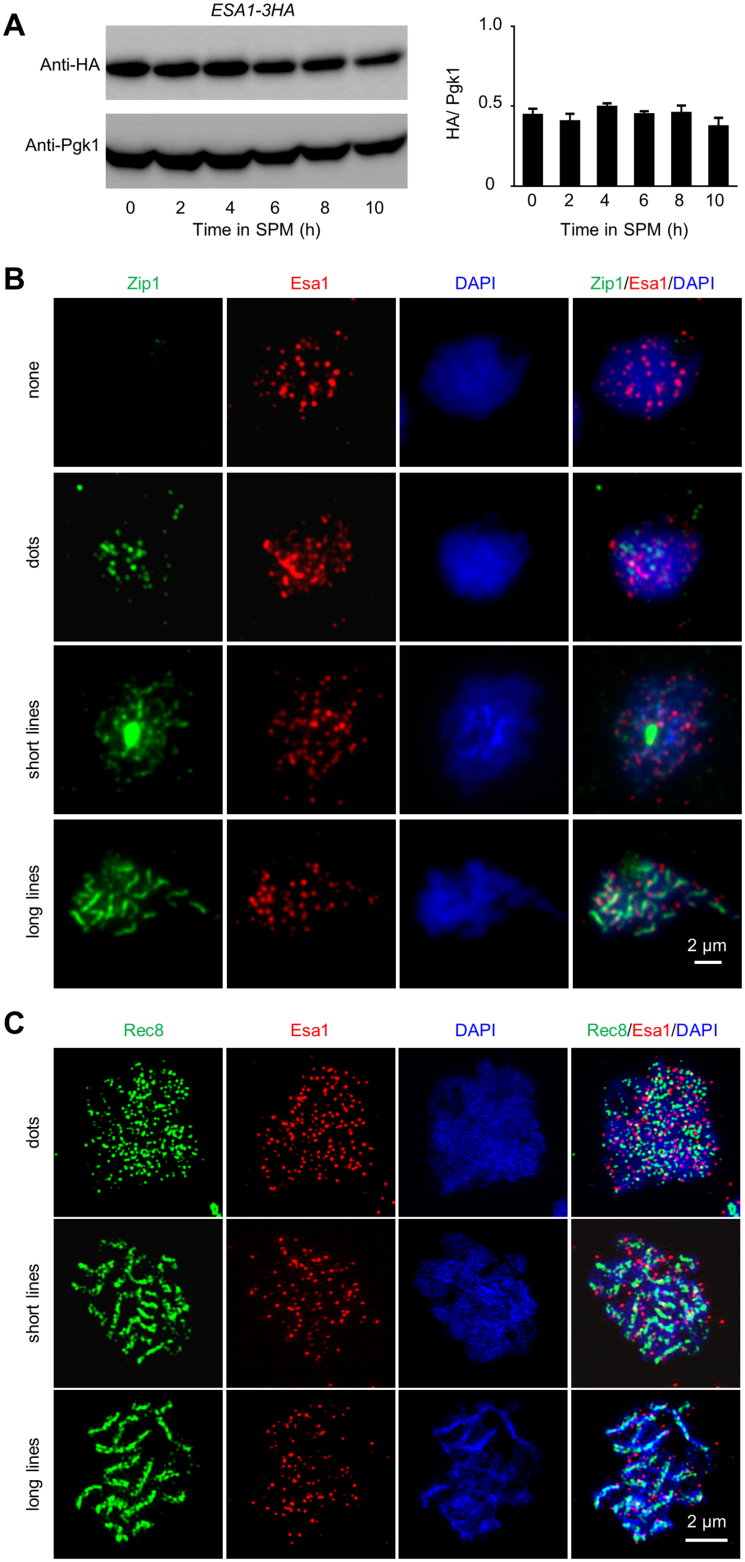
Esa1 is constitutively expressed and localizes on chromatin loops during meiosis. (**A**) Western blot to show constitutive expression of Esa1 during meiotic prophase I. The Esa1 was tagged by 3HA and its expression was detected by antibodies against HA (left, top). The expression of Pgk1 was used as a quantitative control (left, bottom). Quantification of the expression of Esa1 from four biological replicates (right). Error bar, SE. (**B**) Co-immunostaining of Esa1 and Zip1 (a component of synaptonemal complex central element) to show the localization of Esa1 during meiotic prophase I under an epifluorescence microscope. (**C**) Co-immunostaining of Esa1 and Rec8 (a component of meiotic chromosome axial element) to show the localization of Esa1 during meiotic prophase I under a structured illumination microscope (SIM). Samples were collected at 3, 4 and 5 h in SPM (B and C). Scale bar, 2 μm (B and C).

To get insights into the role of Esa1 during meiosis, its expression was first examined. For this purpose, endogenous Esa1 was tagged with 3xHA (hemagglutinin) at its C-terminus (Methods). This tagged *ESA1-3HA* strain grew normally in vegetative media and completed meiosis as wild type (WT) (Supplementary Figure S1A–C). It has been reported that Esa1 localizes on chromatin as foci independent of cell cycle during mitosis (72,73). Our western blot showed Esa1 was constitutively expressed during meiosis (Figure 1A). Its chromatin localization was examined during meiotic prophase I. As shown, Esa1 localized on the surface spread chromatin as foci at different stages based on staining the synaptonemal complex component Zip1 (Figure 1B). Only 3.41% Esa1 foci (112 among 3281 Esa1 foci from 104 nuclei) overlapped with Zip1 signal (Figure 1B). Moreover, only 1.8% Esa1 foci (23 among 1381 Esa1 foci from 60 nuclei) overlapped with Rad51 foci. The detailed localization of Esa1 was further investigated under a structured illumination microscope (SIM) by co-staining with an important axis component Rec8. As observed under an epifluorescence microscope, Esa1 localized on chromatin during prophase I with numerous foci and mostly (97.02% among 5100 Esa1 foci from 102 nuclei) outside the Rec8 stained chromosome axes (Figure 1C and Supplementary Figure S1D). Taken together, these results reveal that Esa1 is constitutively expressed and localizes on chromatin loops during meiosis.

### Esa1 is required for normal meiosis

**Figure 2.**
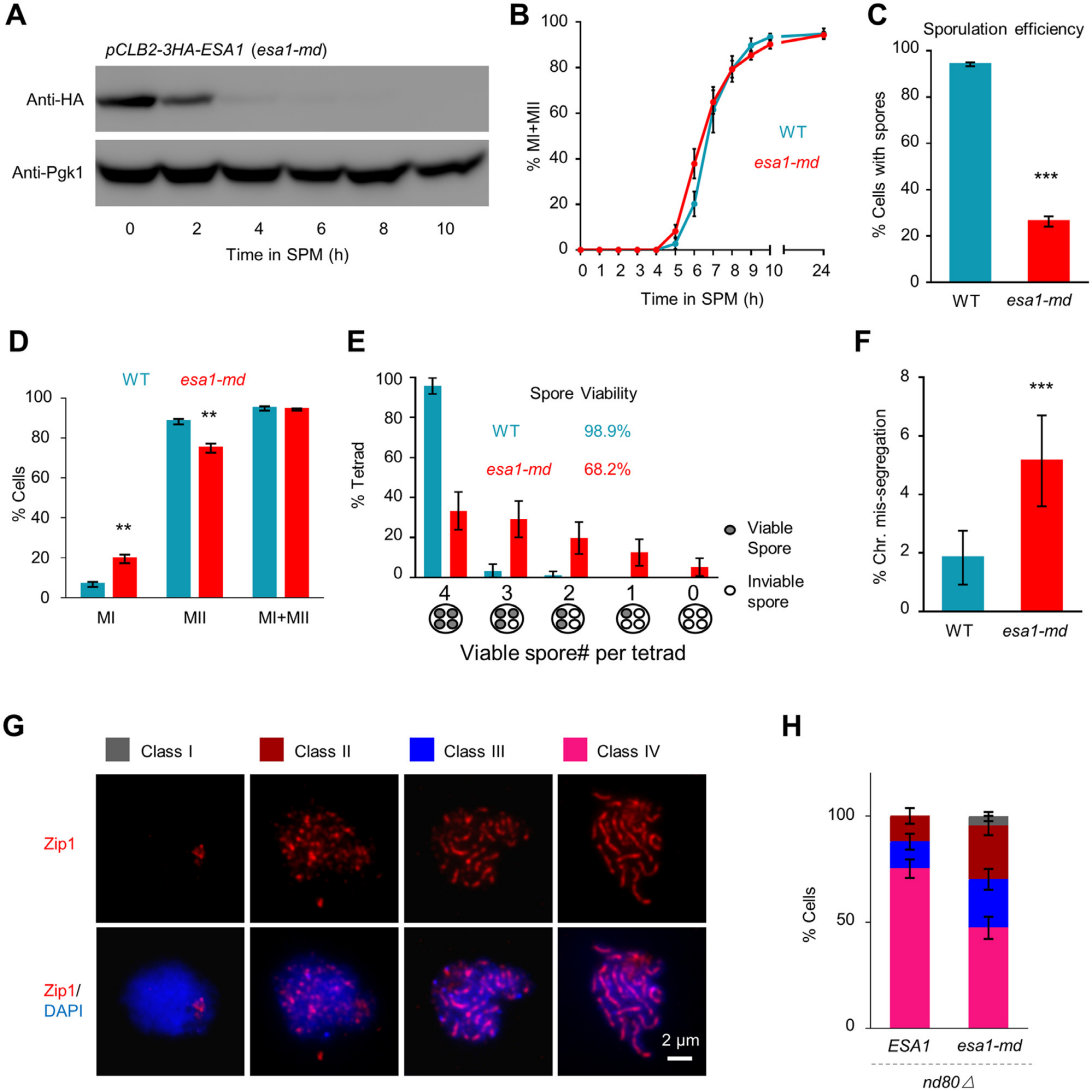
Esa1 is required for normal meiosis. (**A**) Barely detectable Esa1 in SPM after 2 h in *esa1-md* (*pCLB2-ESA1*) via Western blot. (**B**) WT like time course in meiosis-specific depletion of Esa1. Experiments were repeated 4 (WT) or 5 times (*esa1-md*), and >200 nuclei were examined at each time point in each experiment. (**C**) Significantly decreased sporulation efficiency in *esa1-md* strain. Experiments were repeated twice for both WT and *esa1-md* mutant; >200 cells were examined at 24 h in SPM in each experiment. Error bar, SE. (**D**) Increased frequency of nuclei only completed MI and decreased frequency of nuclei completed MII, however, similar frequencies of nuclei completed at least MI. Experiments were repeated 4 (WT) and 5 times (*esa1-md*), and >200 nuclei were examined at 24 h in each experiment. Error bar, SE. (**E**) Decreased spore viability and frequency of tetrads with four-viable spores accompanied by increased frequencies of tetrads with <4-viable spores in the *esa1-md* mutant. Totally 95 (WT) and 96 (*esa1-md*) tetrads were assayed (same WT data as in Figure S1C). Error bar, 95% confidence interval. (**F**) The significantly increased frequency of chromosome mis-segregation in *esa1-md* mutant. Totally 815 (WT) and 777 (*esa1-md*) tetrads were examined; Error bar, 95% confidence interval. (**G**) Partially impaired synapsis in the *esa1-md* mutant. Synapsis was monitored by immunostaining Zip1 (the central element of SC) in *ndt80Δ* background at 10 h in SPM when all nuclei should be arrested at pachytene. Scale bar, 2 μm. (**H**) Quantification of the frequencies of nuclei with different Zip1 morphologies (Class I–IV for none, dots, short lines, and long lines, respectively) as shown in (G). Totally 300 (WT) and 356 (*esa1-md*) nuclei were examined. Error bar, 95% confidence interval. ** (*P* < 0.01); *** (*P* < 0.001); Two proportion z-test (C, D, F).

Given *ESA1* is essential for vegetative growth, to investigate its roles in meiosis, we replaced its native promoter with the *CLB2* promoter, which is active during mitosis but is strictly suppressed during meiosis (74,75), and constructed a meiosis-specific depletion allele *pCLB2-ESA1*, hereafter *esa1-md*. The *esa1-md* yeast grows normally and the Esa1 protein level decreased dramatically upon entering meiosis and was almost undetectable after 2 h in SPM (Figure 2A).

Both WT and *esa1-md* strains were synchronized and induced to enter meiosis. Samples were collected every hour from SPM and fixed for DAPI staining to examine nuclear division. Esa1 depletion has little influence on meiotic nuclear division temporally and ∼94% of nuclei completed at least MI as in WT (Figure 2B). In WT, ∼94% of nuclei sporulated. However, the sporulation frequency was dramatically decreased to ∼26% in *esa1-md* (Figure 2C). Detailed analyses showed that in WT, 89% of nuclei completed MII and only 6% of cells showed arrest after MI. In *esa1-md*, the frequency of nuclei completed MII slightly decreased to 75% and the frequency of nuclei only completed MI increased to ∼20% (Figure 2D). These results suggest that (i) *esa1-md* can complete MI as efficiently as in WT, (ii) a majority of *esa1-md* nuclei can also complete MII although it is less so efficient than WT, (iii) among nuclei completed MII, only a small fraction (26%/75% = 35%) sporulates, which further indicates Esa1 depletion severely impairs sporulation.

To test whether those spores are viable, tetrads were dissected onto YPD plates and incubated at 30°C. After 3 days, the viable spore patterns and spore viability were calculated. In WT, up to 95% tetrads have four viable spores and up to 98% spores are viable (Figure 2E). However, the frequency of tetrads with four viable spores was significantly decreased to 33% in *esa1-md*, and the frequencies of tetrads with different numbers of inviable spores were increased. As a result, spore viability was decreased to ∼68% in *esa1-md* (Figure 2E).

In WT, inviable spores mainly result from aberrant chromosome segregation (76). However, this observed spore viability pattern of *esa1-md* is incompatible with MI homolog nondisjunction, which would produce higher frequencies of tetrads with zero- and two-dead spores. To test whether chromosome missegregation is increased in *esa1-md*, segregation error frequency of chromosome IX was investigated by spore-specific fluorescence assay (Materials and Methods) (77). In this assay, spore-specific promoter driven YFP (*P^YKL050c^-YFP*) and RFP (*P^YKL050c^-RFP*) were integrated near the centromere of the two homologous chromosome IX, respectively. Proper chromosome segregation generates tetrads, among which two spores bear yellow fluorescence and the other two bear red fluorescence. Tetrads with mis-segregated chromosome IX show aberrant patterns of yellow/red fluorescence (Supplementary Figure S2). In *esa1-md*, ∼5% tetrads had chromosome IX segregation errors, which is significantly higher than that in WT (1.8%; Figure 2F). Furthermore, it seems that most chromosome mis-segregation occurs during MII other than MI (Supplementary Figure S2), which is consistent with the observed spore viability pattern (Figure 2E).

Homolog synapsis mediated by the synaptonemal complex (SC) is a hallmark of meiosis. To examine whether *esa1-md* influences synapsis, we analyzed the morphology of SC central element Zip1 in the *ndt80Δ* background where cells can be arrested at pachytene. In wild type, at the beginning of meiosis, no Zip1 signal can be detected (class I). Along with the progression of prophase I, Zip1 first appears as dotty (class II), and gradually grows as partially elongated (class III) and then fully elongated (class IV) structures (Figure 2G). At 10 h in SPM, almost all nuclei should reach and be arrested at pachytene in the *ndt80Δ* background for both WT and *esa1-md*. In WT, all nuclei have visible Zip1 signal and ∼75% of nuclei have fully elongated Zip1 (Figure 2GH). However, only ∼45% of nuclei have fully elongated Zip1 in *esa1-md* mutant (Figure 2GH). Moreover, more nuclei have Zip1 polycomplex structure (∼26% in *esa1-md* versus ∼9% in WT), which further indicates SC defect in *esa1-md* (Supplementary Figure S3).

These results indicate that Esa1 is required for normal meiosis and its depletion leads to multiple meiotic defects including synapsis, chromosome segregation, sporulation and spore viability.

### Meiosis-specific depletion of Esa1 decreases crossover frequency

**Figure 3.**
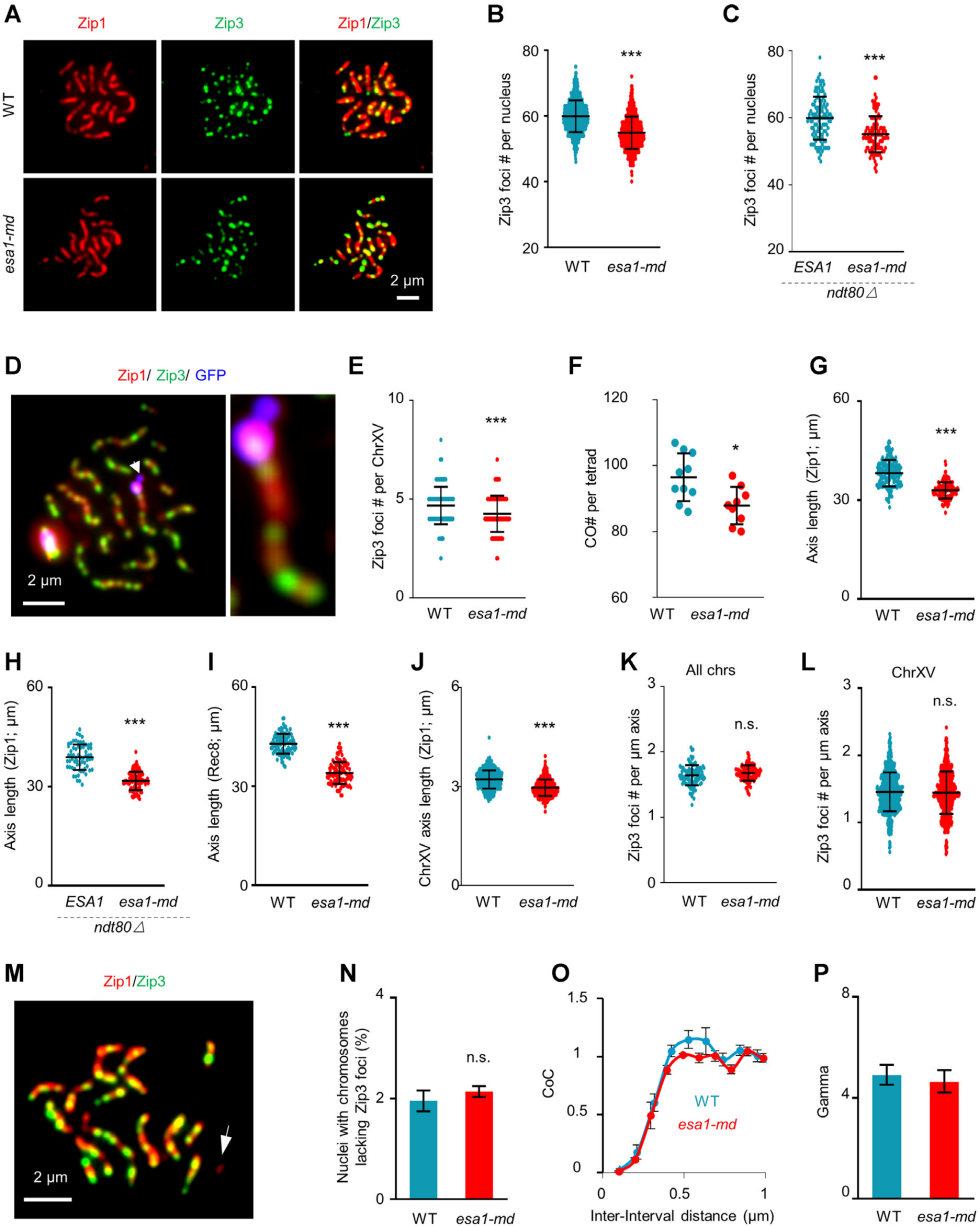
Esa1 regulates chromosome axis length and consequently CO frequency. (**A**) Representative images to show Zip3 foci (green) in pachytene nuclei (judged by Zip1 staining, red) for WT and *esa1-md* strains. Scale bar, 2 μm. (**B**) In *esa1-md*, the number of Zip3 foci per nucleus was decreased to ∼91% of WT. Experiments were done three times in both WT and *esa1-md* strains with > 200 nuclei examined in each experiment. A significantly lower number of Zip3 foci was observed from each experiment. Data from three experiments were pooled and plotted. *N* = 722 (WT) and 774 (*esa1-md*) nuclei. (**C**) Decreased Zip3 foci in *esa1-md* in the *ndt80Δ* background. *N* = 114 (WT) and 107 (*esa1-md*) nuclei. (**D**) An representative image to show the *lacO*/LacI-GFP (blue) marked chromosome XV and Zip3 foci (green) in a pachytene nucleus (judged by Zip1 staining, red). (**E**) In *esa1-md*, the number of Zip3 foci per chromosome XV was decreased to ∼91% of WT. Data from three experiments were pooled and plotted. *N* = 639 (WT) and 661 (*esa1-md*). (**F**) In *esa1-md*, the number of COs detected by genomic re-sequencing of the four spores from a single tetrad was decreased to ∼91% of WT. *N* = 10 (WT) and 9 (*esa1-md*) tetrads. (**G–J**) Shorter chromosome axis length in *esa1-md* as measured nucleus-widely by staining Zip1 (G, H) or Rec8 (I), or on chromosome XV (J). The number of nuclei examined in (G-J): 176, 65, 102 and 639 (WT), 135, 107, 102 and 661 (*esa1-md*). The same set of nuclei used in (E) and (J). (**K, L**) Unaltered number of Zip3 foci per micron of axis length because of the proportionally decreased number of Zip3 foci and axis length either genome-widely (K) or on chromosome XV (L). *N* = 103 (WT) and 125 (*esa1-md*) in (K). The same set of nuclei used in (E), (J) and (L). Error bars, SD (B, C, E–L). (**M**) An representative image to show a small chromosome lacking Zip3 foci (arrow). (**N**) Unaltered frequency of nuclei with chromosomes lacking Zip3 foci. At least 150 nuclei were analyzed for each strain in each experiment. In all cases, it is always short chromosomes lacking Zip3 foci. Error bar, SE from three independent experiments. Two proportion z-test. (**O, P**) CO interference was maintained at the WT level in the *esa1-md* mutant. CO interference was analyzed by CoC curves (**O**) or Gamma distribution with Zip3 focus data for chromosome XV (**P**). Error bar, SE from three independent experiments, about 200 bivalents were analyzed for each experiment (O); 95% confidence interval (P), sample sizes are as in panel (O). Samples used for cytological analysis in *NDT80* background were collected at 5h in SPM. Samples used for cytological analysis in *ndt80Δ* background were collected at 10h in SPM. * (*P* < 0.05); ** (*P* < 0.01); *** (*P* < 0.001); n.s., not significant (*P*> 0.05); Student's *t*-test except specifically indicated.

During mitosis, Esa1 and its orthologs acetylate histones and play a critical role in DNA damage repair (49,50,52). During meiosis, in each nucleus, a large number of programmed DSBs form and then are repaired primarily as crossovers or non-crossovers with its homolog chromosome as the template (2,3). The above results showed that Esa1 plays multiple roles during meiosis, so we further investigated whether Esa1 also functions in meiotic recombination. During budding yeast meiosis, Zip3 is the earliest marker of crossover recombination (24,78) and can be easily visualized by immunostaining the surface spread pachytene nuclei (Figures 3A and Supplementary Figure S4A). In WT pachytene, as previously reported there are averagely ∼60 Zip3 foci per nucleus (24,78). However, in *esa1-md*, only ∼55 Zip3 foci per nucleus were observed, which is a ∼10% decrease compared with WT (Figure 3AB). The Myc tag at Zip3 C-terminus did not affect its function (Supplementary Figure S1). The decrease of Zip3 focus number in *esa1-md* was also observed in a *ndt80Δ* background, which arrested nuclei at pachytene and thus excluded the possible effect of Zip3 turnover (Figure 3C). To further confirm this result, we also measured the number of Zip3 foci on chromosome XV, which is specifically labeled and distinguished by the *lacO*/LacI-GFP reporter (Methods) (12). The number of Zip3 foci on chromosome XV also had a ∼10% decrease in *esa1-md* compared with that in WT, and this indicates that each chromosome probably has the same degree of decrease for Zip3 focus number (Figures 3D, E and Supplementary Figure S4B).

CO recombination can also be detected genetically when parents have proper nucleotide polymorphisms. To further confirm Esa1 regulating CO level, *esa1-md* was introduced into S96 and YJM789 strains, which have ∼50 000 SNP markers averagely spaced at ∼80 bp and uniformly distributed on all chromosomes (13,79). COs are recognized by reciprocal exchange of SNP markers. Consistent with previous results (13,79), averagely each WT tetrad has ∼96.5 COs. However, each *esa1-md* tetrad only has ∼87.9 COs (Figures 3F and Supplementary Figure S5), and consistent with cytological observation, it is a ∼10% decrease compared with WT. Therefore, Esa1 is required for the efficient formation of meiotic COs.

### Esa1 regulates CO frequency via modulating chromosome axis length

Since the number of CO recombination is largely determined by chromosome axis length (34), it would be interesting to examine whether Esa1 regulates CO frequency via modulating axis length or via some other ways. For this purpose, the total length of SC per nucleus at pachytene was measured in surface spread nuclei (Figure 3A). Compared with WT, the SC length per *esa1-md* nucleus has a ∼13% decrease (Figure 3G; 33 μm in *esa1-md* versus 38 μm in WT). The similar decrease of axis length in *esa1-md* was also observed in a *ndt80Δ* background (Figure 3H). Since Esa1 depletion causes SC defects, to exclude the possibility that some nuclei may have incomplete SC thus show shorter SC length, we also measured the length of Rec8 lines (a key component of chromosome axis). Consistently, axis length as revealed by Rec8 staining was similarly decreased as shown by Zip1 staining in the *esa1-md* mutant (Figure 3I; 34 μm in *esa1-md* vs 43 μm in WT). The HA tag fused to Rec8 C-terminus did not affect the function of Rec8 in *esa1-md* (Supplementary Figure S1EF). Moreover, chromosome XV also showed a ∼10% decrease in SC length (Figure 3J). Consistent with the nearly proportional decrease of chromosome axis length and CO number, the number of COs per unit of axis length was maintained unaltered (Figure 3K, L). These results support Esa1 depletion leads to decreased chromosome axis length, which further results in a proportional decrease of CO frequency.

COs are tightly controlled (12,23). Firstly, to ensure proper homologous chromosome segregation, each pair of homolog obtains at least one CO, the so-called obligatory CO. Secondly, when more than one CO exist on a pair of homologs, they tend to stay far away from each other. This phenomenon is known as CO interference, which implies the existence of one CO suppresses the occurrence of other COs nearby. The existence of obligatory CO is reflected by the low frequency of chromosomes lacking COs. Our observations showed that Esa1 depletion decreased CO frequency but it did not increase the frequency of nuclei or chromosomes (judged by Zip1 lines) absence of CO-related Zip3 foci (Figure 3M, N). Therefore, Esa1 depletion does not impair the occurrence of obligatory CO.

To investigate whether the decreased number of COs result from altered CO interference, we mapped positions of Zip3 foci on each chromosome XV in WT and *esa1-md* from >600 nuclei for each strain. The CoC (coefficient of coincidence) curve method is the classic and most accurate measurement of CO interference (Methods) (12). To calculate CoC curves, chromosome XV was normalized to ‘1’ and divided into multiple intervals as previously described (12). Each Zip3 focus was then assigned to a given interval on a given chromosome XV based on its position on that chromosome. For each pair of intervals, the observed frequency of double COs was calculated as the frequency of chromosomes bearing Zip3 foci in both intervals, the predicted frequency of double COs was calculated as the product of the CO frequencies in the two individual intervals, and the ratio of observed/predicted frequency of double COs was the CoC in this interval pair. A CoC curve was obtained by plot CoC values from all interval pairs against corresponding inter-interval distances (the distance between the midpoints of the two involved intervals). The two CoC curves from WT and *esa1-md* were almost overlapped, and there was no difference for the part of CoC from 0 to 1 which indicates the strength of CO interference (Figure 3O). We also did the best fit of the inter-adjacent Zip3 distances to gamma distribution to calculate the shape parameter, which is usually used as another convenient indicator for CO interference (12). Again, no significant difference was detected between WT and *esa1-md* (Figure 3P). Therefore, Esa1 depletion does not impair the formation of obligatory CO or the strength of CO interference, which indicates Esa1 depletion does not affect the basic CO patterning process *per se*, although it decreases chromosome axis length and consequently decreases CO frequency.

**Figure 4.**
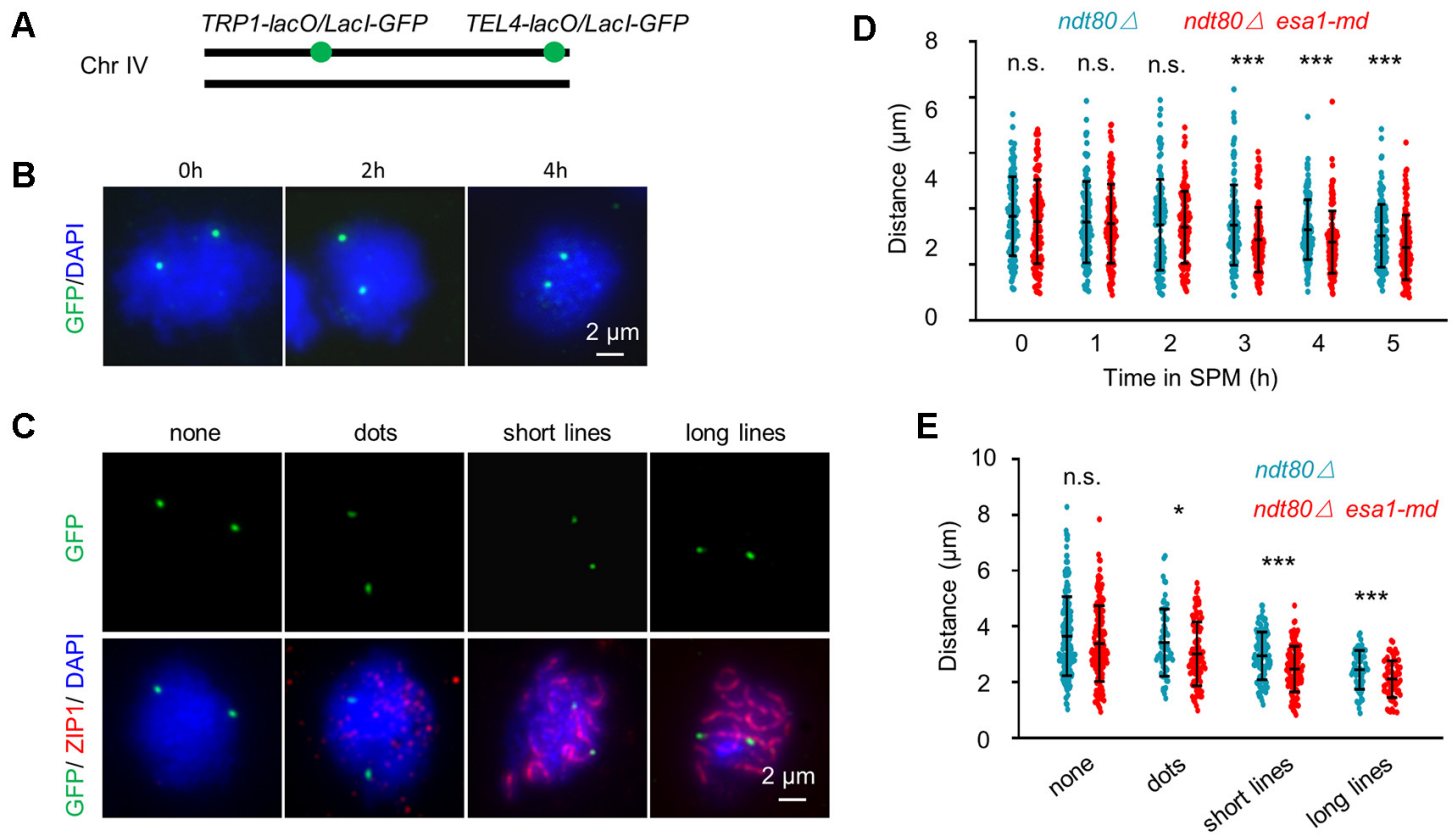
Esa1 regulates chromosome compaction from early prophase I. (**A–C**) Cartoon (A) and representative images (B, C) for examination of the distance between two GFP loci on chromosome IV in spread nuclei in *ndt80Δ* background. Scale bar, 2 μm. (**D**) Quantification of (B). 118, 115, 115, 118, 150 and 131 (WT); 128, 114, 106, 120, 126 and 116 (*esa1-md*) nuclei were examined from 0 to 5 h in SPM, respectively. Data from two independent experiments were pooled. (**E**) Quantification of (C). 208, 62, 106 and 65 (WT); 147, 88, 114 and 64 (*esa1-md*) nuclei were examined for each type. Error bars, SD. * (*P* < 0.05); *** (*P* < 0.001); n.s., not significant (*P*> 0.05); Student's *t*-test.

To reveal the mechanistic insight on how reduced chromosome axis length decreases CO frequency, chromosome compaction during early prophase I and meiotic recombination process were further investigated. Since it is hard to measure chromosome axis length/compaction before pachytene, we measured the distance between two loci marked with GFP spots on only one of the two chromosome IV as previously performed (Figure 4A–E; 57,60,61). When measured in a standard synchronized culture, the distances between the two GFP spots were not significantly different between WT and *esa1-md* mutant from 0 to 2 h in SPM when nuclei were at pre-meiotic DNA replication (Figure 4BD). However, from 3 h in SPM when most nuclei were at leptotene and DSBs were abundant, the distances showed ∼15% shorter in *esa1-md* mutant than WT (Figure 4BD). To exclude possible synchronization difference and more accurately compare chromosome compaction difference between WT and *esa1-md* mutant, the distances between the two GFP spots were also measured and compared in nuclei at different stages according to Zip1 morphologies (61). This analysis confirmed that chromosomes were more compacted from leptotene (Zip1 appears as multiple dots) in *esa1-md* mutant than WT (Figure 4C, E). Similar chromosome compaction was observed between WT and *esa1-md* before leptotene (without Zip1 signal) and in synchronized culture from 0 to 2 h in SPM (Figure 4CE). This probably because during this period a high level of Esa1 still existed in *esa1-md* mutant (Figure 2A), meiotic chromosome axes are not well formed (65), or possible difference in the regulation of chromosome compaction between mitosis and meiosis (61).

**Figure 5.**
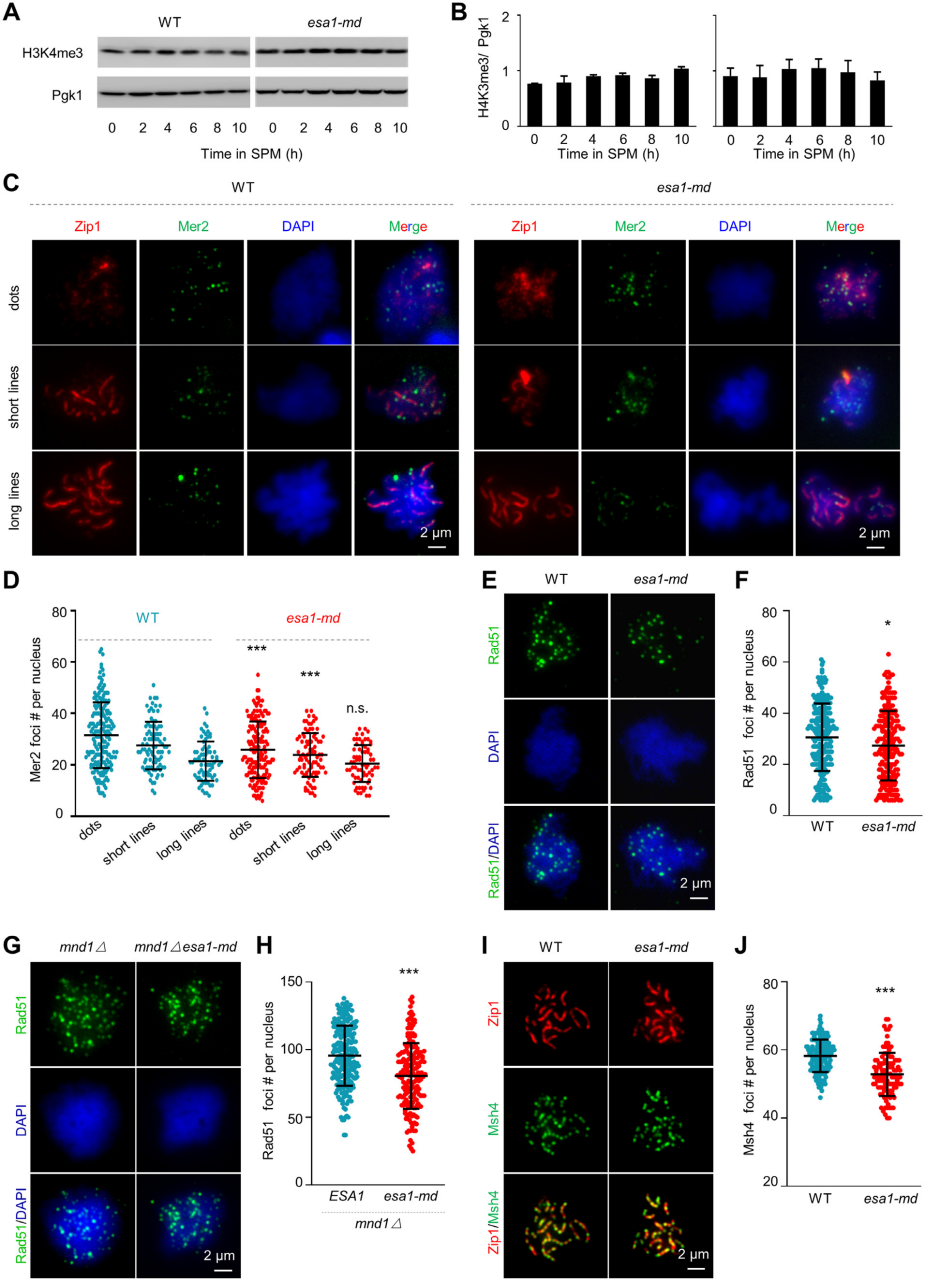
Esa1 regulates meiotic DSB and recombination frequencies. (**A**) Detection of H3K4me3 by Western blot. Samples were collected from synchronized cultures at different time points in SPM. (**B**) Quantification of (A). Error bar, SE from three experiments. (**C, D**) Representative images (C) and quantification (D) of Mer2 foci (green) in nuclei at different stages (judged by Zip1 morphology, red). The number of Mer2 foci was at the highest level in leptotene nuclei (‘dots’) and gradually decreased along with meiosis progression in both WT and *esa1-md*. However, the *esa1-md* mutant showed ∼15% decrease in Mer2 focus number in both leptotene and zygotene nuclei (‘dots’ and ‘short lines’) compared with WT. Mer2 foci were still observed in pachytene nuclei (‘long lines’) with comparable numbers in WT and *esa1-md*. Mer2, Rec114, and Mei4 foci have already been observed on pachytene chromosomes in previous studies although their roles are unclear after DSB formation (55,119,120). Sample size (from left to right), *N* = 189, 90, 73, 163, 85 and 68 nuclei. (**E, F)** Representative images (E) and quantification (F) of Rad51 foci (green) in spread nuclei (blue). Samples collected at 4 h in SPM when most nuclei were at zygotene with maximal Rad51 foci. *N* = 234 (WT) and 214 (*esa1-md*) nuclei. (**G, H**) Representative images (G) and quantification (H) of Rad51 foci (green) in spread nuclei (blue) in *mnd1D* background. Samples collected at 10h in SPM. *N* = 218 (WT) and 179 (*esa1-md*) nuclei. (**I, J**) Representative images (I) and quantification (J) of Msh4 foci (green) in pachytene nuclei. Samples collected at 5 h in SPM. *N* = 164 (WT) and 106 (*esa1-md*) nuclei. Scale bar, 2 μm. Error bar, SD (D, F, H, J). * (*P* < 0.05); ** (*P* < 0.01); *** (*P* < 0.001); Student's *t-*test.

Meiotic recombination initiates from Spo11 complex mediated DSBs (80). Efficient DSB formation requires COMPASS mediated H3K4 tri-methylation (H3K4me3) and Rec114-Mer2-Mei4 (RMM) complex (81–85). In WT, nearly constant levels of histone H3K4me3 were observed by western blot with samples at different time points collected from synchronized meiosis culture (Figure 5AB). Comparable H3K4me3 levels were also observed in *esa1-md* mutant as in WT (Figure 5A, B). Given the chromosome axis localized RMM is required for efficient DSB formation, the number of Mer2 foci along chromosomes in *esa1-md* mutant was also investigated. Interestingly, this mutant with shorter axes showed ∼15% decrease in Mer2 focus number in both leptotene and zygotene nuclei compared with WT (Figure 5C, D, ‘dots’ and ‘short lines’). Consequently, similarly reduced levels of Rad51 foci (a marker for DSB formation and repair) and Msh4 foci (a marker for recombination intermediates) were observed in *esa1-md* mutant (Figure 5E–J). These results suggest that decreased chromosome axis length in *esa1-md* mutant leads to decreased frequency of meiotic DSBs and consequently decreased frequencies of recombination intermediates and COs, probably by decreasing RMM foci required for efficient DSB formation.

### Esa1 depletion does not impair meiotic recombination at *HIS4LEU2* hot spot

To further investigate the role of Esa1 in regulating meiotic recombination, DSBs and recombinant products were analyzed extensively at a well-characterized *HIS4LEU2* hotspot by standard Southern hybridization analysis with proper enzyme digest and a suitable DNA hybridization probe (Supplementary Figure S6A–D) (77,78). DSBs and meiotic recombination were monitored from synchronized meiosis samples. For DSB and CO assay, extracted genomic DNA was digested with the XhoI enzyme. For CO/NCO assay, genomic DNA was digested with XhoI and NgoIV enzymes. DSBs and recombination products were well separated from parent DNA and quantitatively detected via Southern hybridization using a probe for both parent chromosomes (Supplementary Figure S6A–D).

To examine DSBs more accurately, genomic DNA was collected from a *rad50S* strain, which blocks DSB end resection to accumulate DSBs without repair (86). Our results showed that DSBs accumulated to ∼21% of DNA in both WT and *esa1-md* strains in the *rad50S* background (Supplementary Figure S6E). CO and CO/NCO assay results showed that *esa1-md* did not affect CO formation or CO/NCO ratio (Supplementary Figure S6F, G). Therefore, Esa1 depletion does not affect meiotic recombination at the *HIS4LEU2* hotspot. It seems this result is contrary to the globally decreased DSBs and COs. However, it probably reflects the unique behavior of the *HIS4LEU2* hotspot or the possible locus to locus difference (no CO homeostasis at this site; 22).

### Esa1 acetylates histone H4 to regulates chromosome axis length

**Figure 6.**
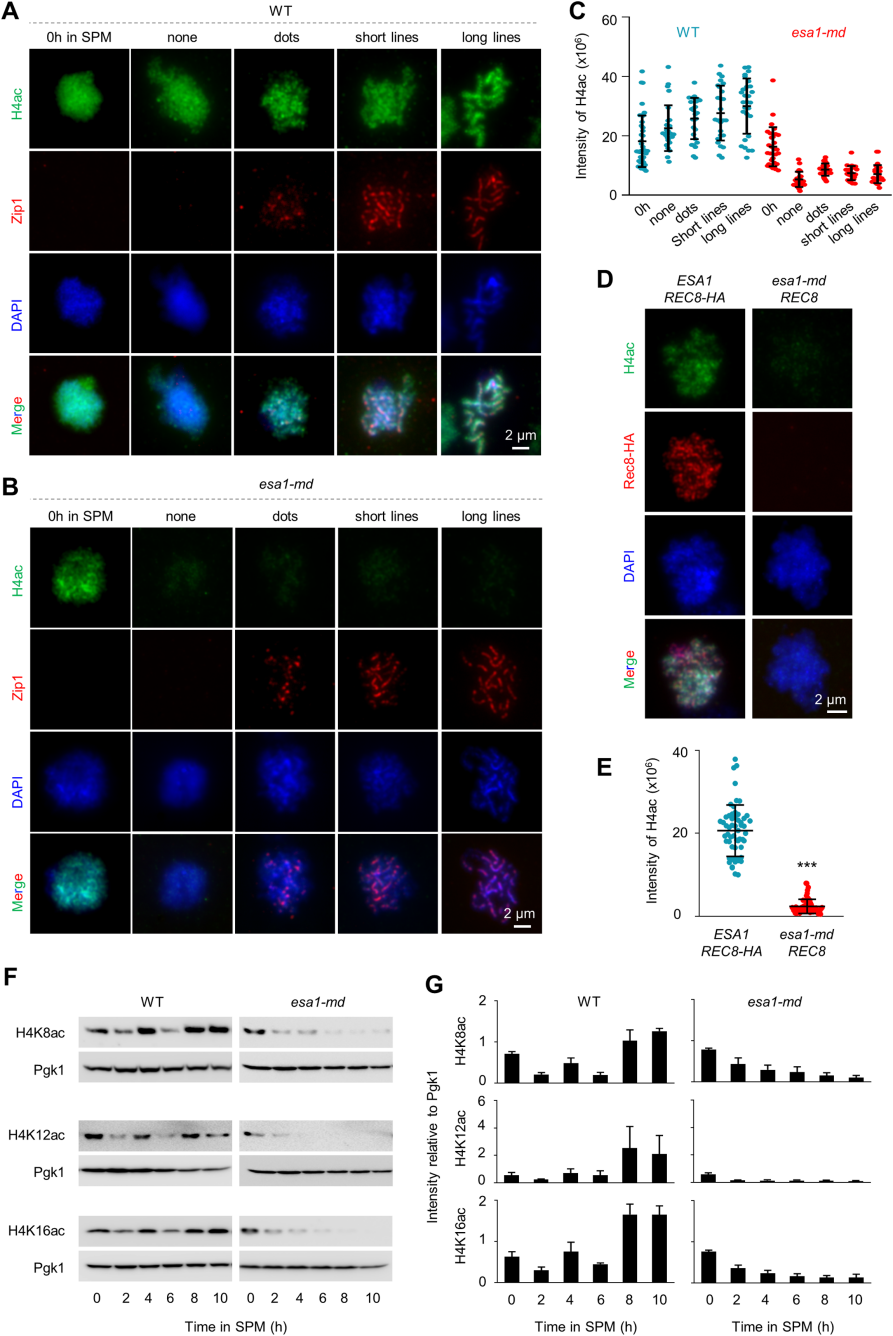
Decreased histone H4 acetylation in *esa1-md* meiotic nuclei. (**A, B**) Representative images of Zip1 and Histone H4 (acetyl K5 + K8 + K12 + K16) staining in WT (A) and *esa1-md* (B) strains. Samples collected at 0 h in SPM were used as control. Nuclei with different Zip1 morphologies and thus at different stages in prophase I were collected from 3 to 5 h in SPM. Scale bar, 2 μm. (**C**) Quantification of histone H4 acetylation levels in (A, B). From left to right, *N* = 39, 27, 28, 26, 28, 29, 25, 26, 26 and 27 nuclei. Error bar, SD. (**D, E**) Decreased histone H4 acetylation in *esa1-md*. Both WT and *esa1-md* samples were collected at 5h in SPM. To accurately compare H4 acetylation between WT and *esa1-md* mutant, nuclei from both strains were spread on the same slide, and WT nuclei were distinguished by staining Rec8-HA with an antibody against the HA tag but the Rec8 was not tagged in *esa1-md*. (E) Quantification of (D). *N* = 57 (WT) and 60 (*esa1-md*) nuclei. Error bar, SD. (**F**) Decreased histone H4 acetylation assayed by Western blot with antibodies against H4K8ac, H4K12ac or H4K16ac. (**G**) Quantification of histone H4 acetylation levels in (F). Error bar, SE from three experiments. *** (*P* < 0.001); Student's *t-*test.

Esa1 is the catalytic subunit of the NuA4 complex which primarily acetylates histone H4 to affect chromatin architecture (45–47), and it may affect chromosome loop/axis organization and thus axis length. Thus, we examined whether Esa1 regulates histone acetylation during meiosis. Using nuclei at 0 h in SPM as a control, strong histone H4 acetylation signal was observed in WT meiotic nuclei during prophase I as shown by immunofluorescence assay (Figure 6A). Very similar histone H4 acetylation levels were observed in nuclei collected from 0 h in SPM in *esa1-md* as in WT. However, only low acetylation levels were detected in meiotic nuclei collected from 3 to 5 h in SPM in *esa1-md* (Figure 6BC). To avoid possible staining difference and get a more accurate comparison of histone H4 acetylation levels between WT and *esa1-md* meiotic nuclei, nuclei from 5 h in SPM in both strains were mixed and spread on the same slide. Since Rec8 was HA tagged in WT but not *esa1-md*, WT nuclei were distinguished from *esa1-md* nuclei stained with an antibody against the HA tag (Figure 6D). The quantification confirmed that histone H4 acetylation levels were significantly decreased during meiosis in *esa1-md* (Figure 6E).

Western blot was further performed to confirm histone H4 acetylation levels in meiosis. Because the antibody recognizing acetylated histone H4 N-terminus used in immunostaining does not work in Western blot, histone H4 acetylation levels were examined at three individual sites: acetylated K8, K12 and K16. The results showed that histone H4 acetylation was significantly decreased at these sites after 2 h in SPM in *esa1-md* strain, which is temporally consistent with the significantly decreased Esa1 (Figure 6F, G). The quick decrease in histone H4 acetylation upon Esa1 depletion indicates an active deacetylation process or histone replacement during meiosis. This result also indicates that Esa1 is indeed the primary acetyltransferase responsible for acetylation of histone H4 N tails during meiosis. A low level of histone H4 acetylation was still observed in the mutant after 2 h in SPM when Esa1 was barely detectable. This may be due to other acetyltransferase work in this process in meiosis (e.g. Hat1 and Sas2 which work in mitosis), a small fraction of acetylated histone H4 has slow turnover or slow replacement by unacetylated ones, or reduced deacetylation activity due to feedback from decreased acetylation. Altogether, the above results indicate that WT level of histone H4 acetylation requires Esa1 in meiosis as in mitosis and raise the possibility that Esa1 regulates chromosome axis length via modulating histone H4 acetylation.

**Figure 7.**
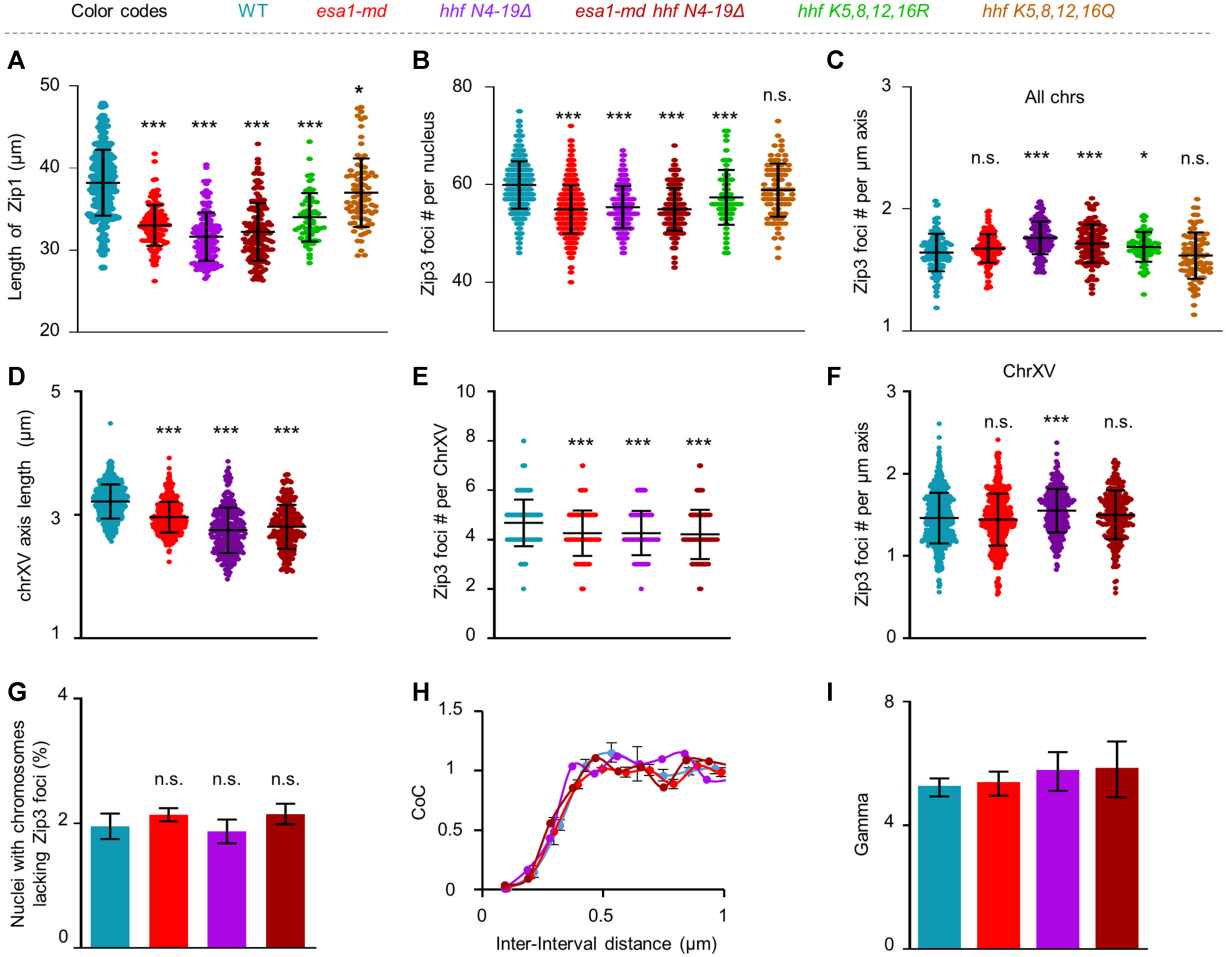
Esa1 regulates chromosome axis length via acetylating histone H4. (**A, B**) Quantification of per-nucleus chromosome axis length (A) and Zip3 focus number (B). Samples were collected at 5h and 6h in SPM. Sample size, from left to right, *N* = 176, 135, 153, 158, 64, 83 (A); N = 722, 774, 209, 196, 64, 88 (B). Error bar, SD. (**C**) The number of Zip3 foci per micron of axis. Sample size, from left to right, *N* = 103, 125, 124, 118, 64, 83. Error bar, SD. Statistic test showed that there were slightly increased Zip3 foci per unit length of axis in *hhf N4-19Δ, esa1-md hhf N4-19Δ* and *hhf K5,8,12,16R* mutants. This probably arise from the fact that chromosomes with shorter axes tend to have higher CO densities seen in various organisms. (**D, E**) Quantification of chromosome XV axis length (D) and Zip3 foci (E). Same sets of nuclei used in (D, E), *N* = 639, 661, 340, 255. (**F**) The number of Zip3 foci per micron of chromosome XV axis. Data from (D, E). Error bar, SD. (**G**) Percentage of nucleus with chromosomes absence of Zip3 foci. At least 150 nuclei were analyzed for each strain in each experiment. Error bars, SE from three independent experiments. (**H**) Coefficient of coincidence (CoC) curves for chromosomes XV. Error bar, SE from at least three independent experiments, about 200 bivalents were analyzed for each experiment. (**I**) Gamma values obtained from best-fit gamma distributions. Error bars, 95% confidence interval. The numbers of chromosome XV analyzed are the same as in panel (**H**). All comparisons are between WT and corresponding mutant; * (*P* < 0.05); *** (*P* < 0.001); n.s, not significant (*P*> 0.05); Two proportion z-test for (G); Student's *t-*test for others. Data for WT and *esa1-md* in this figure from Figure 3.

To confirm the above hypothesis, the *hhf N4-19Δ* (a histone H4 mutant lost N-terminal 4–19 amino acid residues containing Esa1 acetylation sites) mutant was made. This mutant grew slightly slowly and formed smaller clones than WT. During meiosis, this mutant showed similar defects in synapsis and sporulation as the *esa1-md* mutant (Supplementary Figure S7AB). More importantly, as the *esa1-md* mutant, this mutant showed shorter chromosome axis without affecting histone H4 abundance (Figure 7A and Supplementary Figure S7CD). Moreover, in the *hhf N4-19Δ* mutant, there was no observable acetylation and the number of Zip3 foci was also decreased to the same level as in *esa1-md* (Figure 7B, Supplementary Figure S4B and S7E). To exclude the possibility that other modifications but not acetylation of histone H4 N terminus regulate chromosome axis length and CO frequency. Chromosome axis length and CO frequency were further examined in the acetylation- and unacetylation-mimic mutants (*hhf K5,8,12,16Q* and *hhf K5,8,12,16R*, respectively). As expected, the unacetylation-mimic mutant showed decreased chromosome axis length and Zip3 foci, however, the acetylation-mimic mutant showed WT levels of axis length and Zip3 foci (Figure 7A, B and Supplementary Figure S4A). These results suggest that Esa1 and histone H4 acetylation work in the same pathway to regulate chromosome axis length and thus CO frequency. To further confirm this idea, both axis length and CO frequency were also examined in the *esa1-md hhfN4-19Δ* double mutant. As expected, this double mutant showed the same level of chromosome axis length and CO frequency as each single mutant (Figure 7A, B and Supplementary Figure S4B). As a consequence, all mutants have similar CO density per micron axis length as in WT (Figure 7C). The nucleus-wide alterations in both chromosome axis length and CO frequency were further confirmed on chromosome XV, which was labeled by a *lacO*/LacI-GFP marker (Figure 7D–F). Furthermore, the alterations in chromosome axis length and CO frequency did not impair the occurrence of the obligatory CO or the strength of CO interference (Figure 7G–I). All of these results support that Esa1 regulates meiotic chromosome axis length and consequently CO frequency via acetylating histone H4.

### Esa1 regulates chromosome axis length independent of Pds5

**Figure 8.**
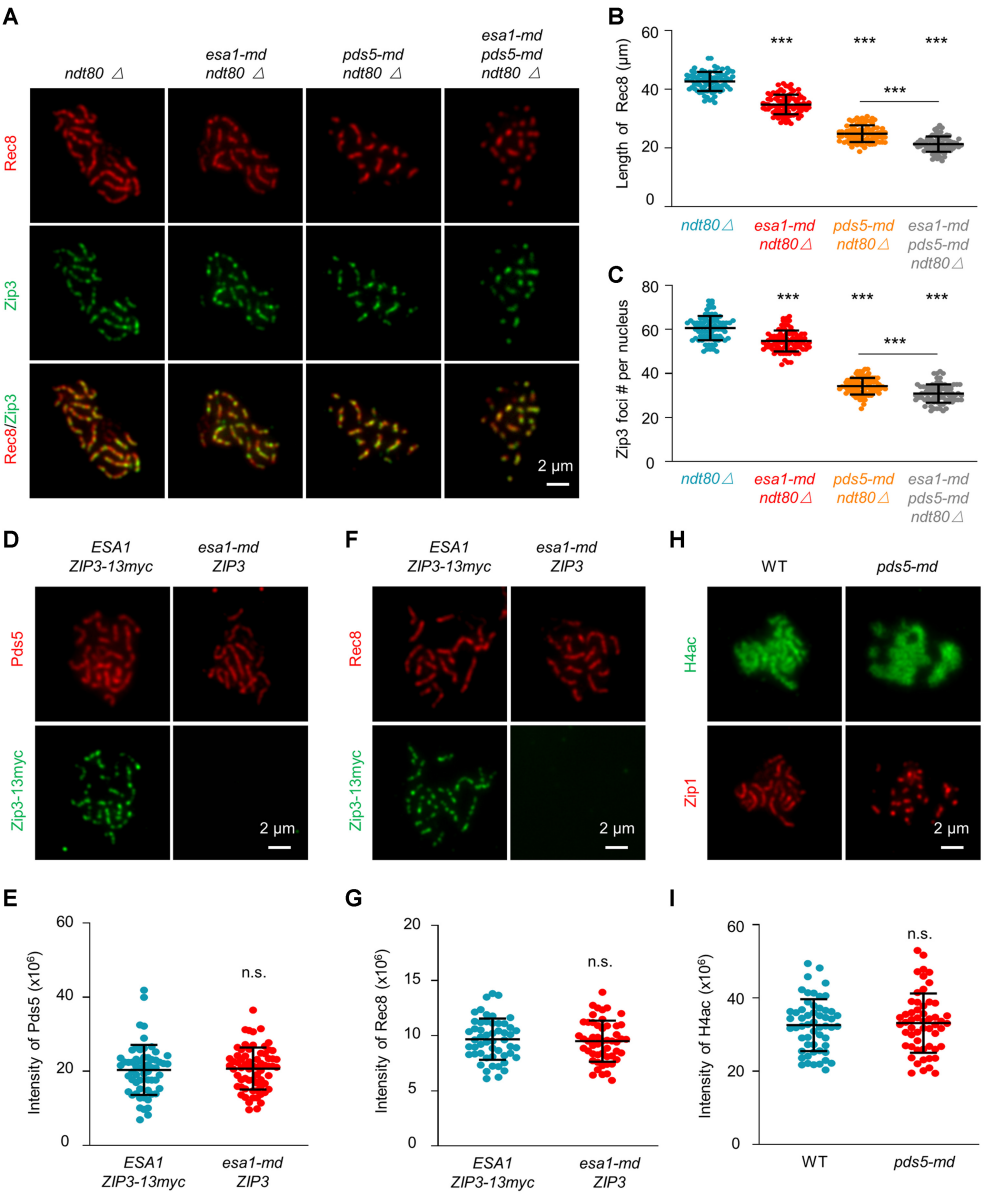
Esa1 regulates chromosome axis length independent of Pds5. (**A**) Representative images to show chromosome axes (Rec8, red) and Zip3 foci (green) in pachytene nuclei. Scale bar, 2 μm. (**B, C**) Quantification of Rec8 length and the number of Zip3 foci from (A). *N* = 81, 87, 84 and 80 nuclei (B, C). (**D–G**) Representative images (D, F) and quantification (E, G) to show Esa1 depletion does not affect the abundance of Pds5 or Rec8. Both WT and *esa1-md* samples were collected at 10h in SPM in *ndt80△* background. To accurately compare Pds5 or Rec8 abundance between WT and *esa1-md* mutant, samples from WT and the mutant were mixed and spread on the same slide. Zip3 was tagged by 13myc in WT but not the mutant, and thus nuclei from these two strains can be easily distinguished after immunstaining. Scale bar, 2 μm. *N* = 57 (WT) and 60 (*esa1-md*) nuclei (E); 52 (WT) and 51 (*esa1-md*) nuclei (G). (**H, I**) Representative images (H) and quantification (I) to show Pds5 depletion does not affect the level of histone H4 acetylation. WT and mutant samples were collected at 5 and 6.5 h in SPM and spread on the same slide and distinguished by their dramatically different Zip1 morphologies. *N* = 55 (WT), 55 (*pds5-md*). All comparisons are between WT and corresponding mutant except specifically indicated. Error bar, SD (B, C, E, G, I). *** (*P* < 0.001); n.s, not significant (*P*> 0.05); Student's *t-*test.

Pds5, a regulator of cohesin, regulates meiotic chromosome axis length in a dosage-dependent manner (58). To examine the relationship between Esa1 and Pds5 in regulating axis length, chromosome axis length in the two single and also the double mutants were measured and compared (Figure 8A). The length of chromosome axes (revealed by Rec8 lines) was decreased to ∼82% and ∼58% of WT level in *esa1-md* and *pds5-md* single mutants, respectively (Figure 8B). Chromosome axis length in *esa1-md pds5-md* double mutant was further decreased to ∼50% of WT level, which is not statistically different from the expected axis length (∼48% of WT level) assuming Esa1 and Pds5 regulate chromosome axis length independently (Figure 8B). Since axis length regulates the number of COs, the number of Zip3 foci per nucleus was similarly decreased to ∼90%, ∼57%, and ∼51% of WT level in *esa1-md*, *pds5-md*, and *esa1-md pds5-md*, respectively (Figure 8C). The observed number of Zip3 foci (31; ∼51% of WT level) is also not statistically different from expected (31; ∼51% of WT level) in the double mutant assuming Esa1 and Pds5 work in two different pathways. These results suggest that Esa1 and Pds5 regulate chromosome axis length (and thus the number of Zip3 foci) independent of each other. Since *pds5* depletion partially disrupt homolog pairing, when axis length and Zip3 focus number were corrected with the number of unpaired homologs as previously described (58), the above conclusion is still valid. This conclusion is further supported by following observations: (i) neither Pds5 nor Rec8 abundance is altered in *esa1-md* mutant and (ii) histone H4 acetylation level is not altered in *pds5-md* mutant (Figure 8D–I).

### Defects in sporulation and chromosome segregation but not chromosome axis length may result from altered transcription program

**Figure 9.**
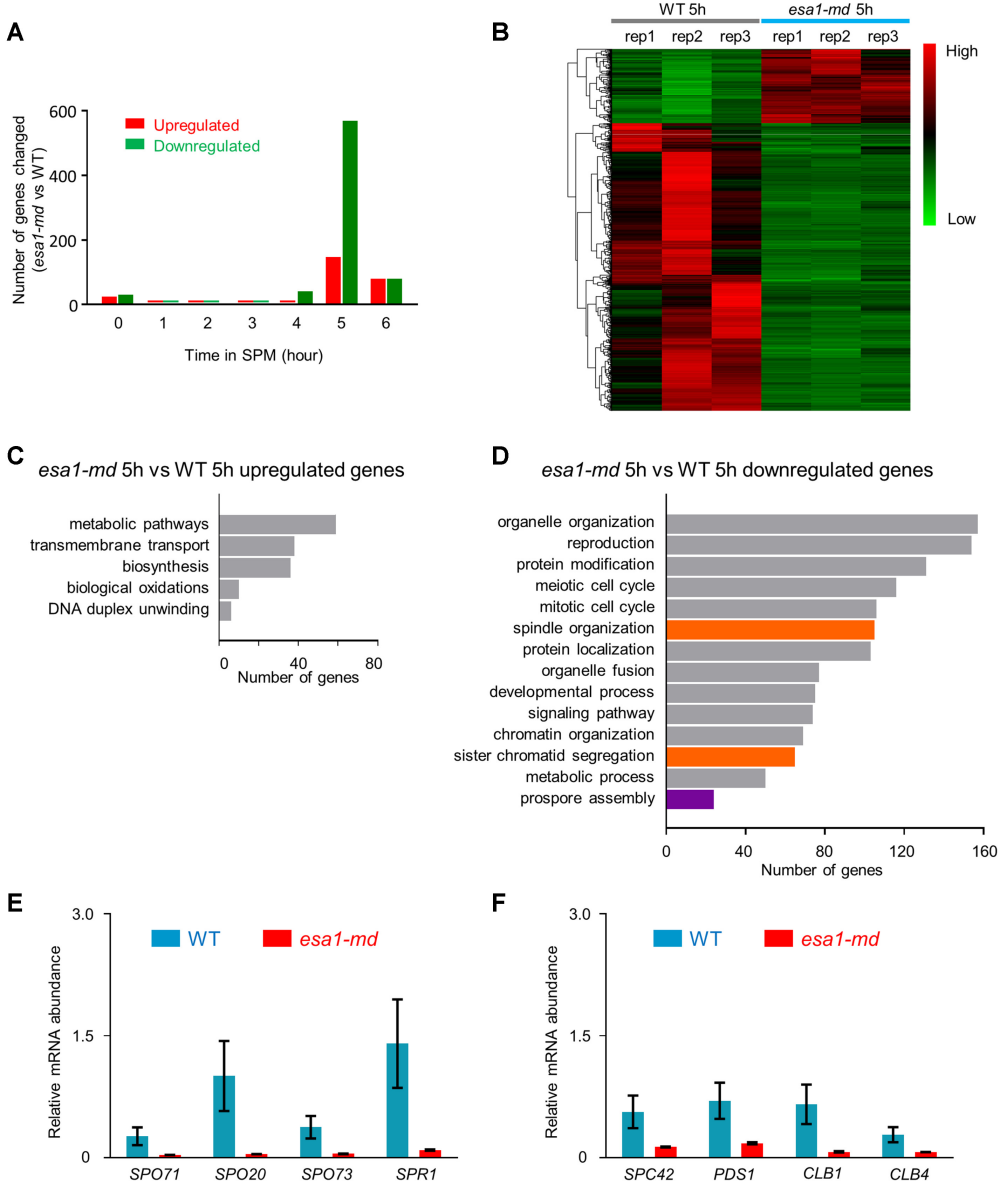
Defects in sporulation and chromosome segregation may result from altered transcription program in the *esa1-md* mutant. (**A**) The number of genes with altered transcription levels in *esa1-md* compared with WT. Sample were collectd from synchronized cultures in SPM at each time point. RNA-seq analyses were performed in biological triplicates. Dfferentially expressed genes were chosed with the criteria: |log_2_(fold change)| ≥ 1 with Benjamini-Hochberg adjusted *P* value <0.05. (**B**) Heatmap of expression profiles for totally 716 differentially expressed genes at 5 h between *esa1-md* and WT. Colors from green to red represent the expression level of genes from low to high. All three replicates were shown. (**C**) Functional enrichment analysis of upregulated genes in *esa1-md* at 5 h in SPM. (**D**) Functional enrichment analysis of downregulated genes in *esa1-md* at 5 h in SPM. (**E, F**) RT-qPCR assays of selected genes downregulated in prospore assembly and spindle organization in WT and *esa1-md* at 5 h in SPM. mRNA abundance was normalized to *ACT1*. Error bar, SE from three repeats.

It is well known that products of early induced meiotic genes (before DSB formation) are involved in regulating chromosome pairing/synapsis and recombination and products of middle induced genes (mainly regulated by a meiosis-specific transcription factor Ndt80 during pachytene) are involved in regulating sporulation (87). Since Esa1 has an important role in regulating transcription during mitotic growth, its possible role in transcription program during meiotic prophase I was also examined. For this purpose, synchronized samples were collected every hour (0–6 h in SPM) for RNA-seq analysis. Compared with WT, transcription abundances of few genes was changed by 2-fold or more during 0–4-h period in SPM (98 among 6809 genes; Figure 9A; Supplementary Table S4). Importantly, it seems that none of the few genes with changed transcription abundance is involved in regulating chromosome structure (Supplementary Table S4). This suggests that alterations in chromosome axis length from early prophase I is less likely resulted from altered transcription program in *esa1-md*.

Significant changes in transcription abundances for a number of genes were observed at 5 h (pachytene) in SPM (716 among 6809 genes; Figure 9AB; Supplementary Table S4). Detailed analyses revealed that transcript abundances for many genes involved in prospore assembly (24 genes) and chromosome segregation (170 genes in spindle organization and sister chromatid segregation) were downregulated (Figure 9C, D; Supplementary Table S4). Altered transcription abundances for representative genes were confirmed by RT-qPCR (Figure 9E, F; Supplementary Table S3). These results suggest chromosome mis-segregation (and thus spore inviability) and sporulation inefficiency in *esa1-md* probably result from defective transcription of genes required for these two processes, respectively.

## DISCUSSION

### Esa1 modulates meiotic chromosome axis length and consequently CO frequency

Histone tail acetylation is one of the best-characterized posttranslational modifications which modulates chromatin structure and gene activity. Acetylation neutralizes the positive charge, which can affect intra- and inter-nucleosome interactions to directly influence higher-order chromatin structure or affect interactions between nucleosome and non-histone proteins to indirectly influence chromatin folding (35,36). Transcription can be influenced by the direct chromatin structure alteration or by altered recruitment/activity of transcription factors (37–39). Both experimental and theoretical studies have shown that among different histones, histone H4 N-terminal tail especially K16 acetylation, plays the most important role in relaxing chromatin (88–91).

In agreement with histone H4 acetylation relaxing chromatin, our results showed that the loss of acetylation on its N-terminal tail, either by direct deletion of the N terminal, meiosis-specific depletion of its acetyltransferase catalytic subunit Esa1, or the mimics of unacetylated status, resulted in shorter meiotic chromosome axes (Figures 3 and 7). Recently, we have shown a cohesin regulator Pds5 dosage-dependently regulate chromosome axis length (58). Our current study revealed that Esa1 regulates axis length independent of Pds5. This raises an interesting question: how Esa1/histone H4 acetylation regulates chromosome axis length?

Meiotic chromosomes are known to be organized as loop/axis structures, and the number of loops per unit length of the axis is highly conserved (27). Therefore, loop size determines axis length which is supported by many observations (29–32,34,92). A loop extrusion model and similar ideas, which is proposed to explain mitotic chromatin loop formation and compaction, has also been used to explain meiotic chromosome organization (34,93–96). Simply, a loop extruding factor holds two loci together and translocates along a chromosome to gradually expand and produce a chromatin loop (95). This process is halted when the extruding factor encounters with another extruding factor or an extrusion barrier, which thus determines the loop size. This model would predict chromatin loop size is negatively correlated with axis length as observed (28–32,34,65,97).

Studies in both mitosis and meiosis suggest that cohesin is the most possible loop extruding factor because cohesin has the expected characters as a loop extrusion factor and mutations affecting cohesin have the exactly expected phenotypes (92,93,98–101). In mammals, CTCF is the most possible extrusion barrier (95). However, CTCF does not exist in yeast and such a barrier has not been found yet.

Our immunostaining results showed that Esa1 primarily localized on chromatin loops with multiple discrete foci (Figure 1 and Supplementary Figure S1). This may indicate that Esa1 mainly acetylates histone H4 on chromatin loops, which is consistent with its known role in transcription. The depletion of Esa1 suppresses transcription thus reduces chromosome bound RNA polymerases, which may work as moving barriers (102). It is possible that RNA polymerases slow down but not stop cohesin movement on chromatin, thus Esa1 depletion speeds up its movement on chromatin with hypoacetylated H4 to produce larger loops and resultant shorter axes. However, Esa1 depletion had little effect on transcription program during early prophase I (Figure 9A). This suggests it is less likely that Esa1 regulates chromosome axis length via altering transcription or RNA polymerases. Another possibility is that hypoacetylation directly affect cohesin, either its activity, translocation speed, or its density along chromosomes. All of these possibilities remain for further investigations.

**Figure 10.**
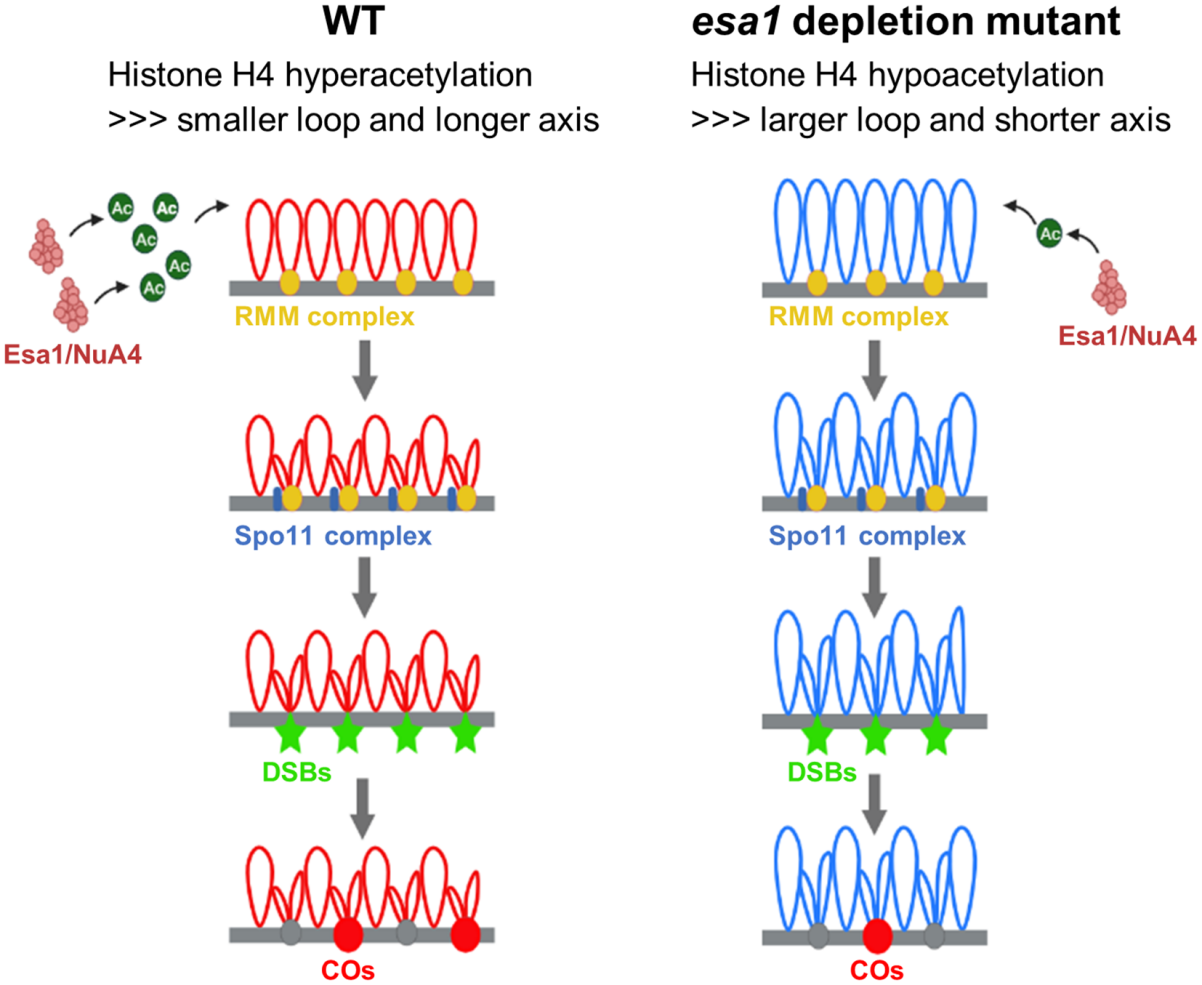
A possible model for Esa1 regulating chromosome axis length and CO frequency. Esa1/NuA4 primarily acetylates histone H4 N tails. In Esa1 depletion mutant, histone H4 is hypoacetylated which results in shorter chromosome axes probably by generating larger loops. Shorter axes have smaller space and accommodate less Mer2/RMM foci on axes. Consequently, less loops can be tethered to Mer2/RMM-rich sites on axes for DSB formation, and ultimately, less COs form.

Chromosome axis length largely determines the number of meiotic DSBs and homologous recombination frequency (28,34). The *esa1-md* has decreased chromosome axis length and roughly proportionally decreased DSB and CO frequencies as in *pds5-md* single and *esa1-md pds5-md* double mutant (Figures 3-5). This indicates that the decreased number of COs results from decreased chromosome axis length. Esa1 depletion does not alter CO interference or the formation of the obligatory CO (Figure 3M–P). Therefore, Esa1 modulates chromosome axis length and consequently recombination frequency but does not affect the basic recombination process *per se* as proposed in *pds5* depletion mutant (58). Further mechanistic investigations reveal that shorter axes decrease Mer2, Rad51, and Msh4 foci but not H3K4me3 levels in *esa1-md* (Figure 5). One possibility is that shorter axes have smaller space accommodating less Mer2/RMM foci on axes, and as a result, only a smaller number of H3K4me3-rich loops can be tethered to Mer2/RMM-axis positions for DSB formation (Figure 10).

CO homeostasis works to maintain CO number less affected at the expense of NCOs when DSB number is altered (12,22,24–26). The current view is that CO homeostasis results from CO interference and its strength depends on the strength of CO interference (12,24,103,104). CO interference spreads along microns of chromosome axes to inhibit further occurrence of COs nearby. Thus, CO frequency on a given chromosome is mainly determined by the interplay between axis length and CO interference distance (28,34,58,104,105). In *esa1-md* mutant, CO interference is not altered (Figures 3OP and 7HI), which indicates CO homeostasis may be unaltered. Moreover, there are proportional decreases in chromosome axis length, DSBs, and COs in this mutant. These results support that decrease in DSBs and COs results from proportionally decreased chromosome axes. However, it would be interesting to test the strength of CO homeostasis by examining COs in a series of *spo11* hypomorphic alleles with decreased DSBs in *esa1-md* background as previously did (22,24).

### Esa1 plays multiple roles during meiosis

Besides its roles in regulating axis length and consequently recombination frequency, *esa1-md* has a significantly decreased number of nuclei with full SC which is accompanied by an increased number of nuclei with polycomplex, increased chromosome segregation errors (especially chromosome mis-segregation during MII), dramatically decreased sporulation efficiency and spore viability (Figure 2 and Supplementary Figure S3). Currently, we cannot exclude the possibility that these defects are the direct consequences of shortened axes. However, it is very less likely. Firstly, many recombination-defective mutants have SC defects and decreased spore viability (106,107), but as described and discussed above, *esa1-md* does not impair the basic recombination process. Secondly, both axis length and recombination frequency only mildly (∼10%) decrease in the *esa1-md*. It is hard to imagine this small alteration impairs any above-mentioned processes so severely for the following reasons. (i) In many organisms, males and females usually have different chromosome axis lengths and thus different CO frequencies, but both sexes do meiosis efficiently (except that human female has CO maturation inefficiency but this is not related with any synapsis defects) (28,34). (ii) Several mutants have moderately altered chromosome axis length, but they do not have obvious defects in homolog synapsis, chromosome segregation, or spore viability (24,57,108,109). (iii) Among different nuclei, chromosome axis lengths and thus the number of COs vary significantly. However, these nuclei can do meiosis very well (29,31,34,110,111). (iv) Mutants of *spo11* hypomorphisms with lower DSBs or *msh4/5* hypomorphisms with lower CO levels than that in *esa1-md* still showed high spore viability (22,112). Therefore, Esa1 functions in multiple processes during meiosis.

The primary targets of Esa1/NuA4 complex are histones. Histone acetylation, especially histone H4K16 acetylation, can regulate interactions of nucleosomes with non-histone proteins (37–39). RNA-seq analyses showed that transcription abundances for a number of middle induced meiotic genes was decreased during middle prophase I (Figure 9B–D). Middle induced meiotic genes are primarily regulated by the transcription factor Ndt80 (87). Ndt80 surveillances DSB repair and regulates pachytene exit (113). mRNA abundances of *NDT80*, *CLB1* (one important Ndt80 target required for spindle formation and thus proper chromosome segregation), and many genes required for prospore assembly were significantly downregulated in *esa1-md* (Figure 9F; Supplementary Table S4) (114). This suggests that defects in chromosome segregation, spore viability, and sporulation efficiency may be the result of altered transcription program when Esa1 is depleted in meiosis. However, it seems that mRNA abundance of *CDC5*, another important Ndt80 target required for double Holliday junction resolution and pachytene exit, was not changed in *esa1-md* (Supplementary Table S4) (115). Consistently, the timing of nuclear division and recombination process are not (or little) affected in *esa1-md* (Figures 2B and S6). Given both *CLB1* and *CDC5* are regulated by Ndt80, it is unclear why decreased *NDT80* transcription downregulates *CLB1* but not *CDC5* in *esa1-md*. One possibility is that *CLB1* and *CDC5* are differentially regulated by moderately decreased Ndt80. Another possibility is that Esa1 can directly regulate *CLB1*.

The status of histone H4K16 acetylation has also been proposed to play a role in the meiotic recombination checkpoint trigged by defective synapsis, which could also promote additional DSB formation(116–118). Further investigations in *esa1-md* could help to figure out whether Esa1 is involved in this process and whether H4K16 acetylation and Ndt80 work in the same checkpoint pathway.

## DATA AVAILABILITY

Data used in the paper are present in the paper and/or the Supplementary Materials. The *esa1-md* whole-genome resequencing data are available at NCBI (SRA Bioproject, accession number PRJNA747839). RNA-seq data are available at NCBI (SRA Bioproject, accession number PRJNA748179).

## Supplementary Material

gkab722_Supplemental_Files
