# Supplementary material for: ESA1 regulates meiotic chromosome axis and crossover frequency via acetylating histone H4
Source: Nucleic Acids Res. 2021 Aug 20;49(16):9353–73. doi: 10.1093/nar/gkab722 (PMC8450111; doi:10.1093/nar/gkab722)
Supplement: gkab722_Supplemental_Files [file gkab722_supplemental_files.zip › Wang et al. Supplementary Information.pdf]

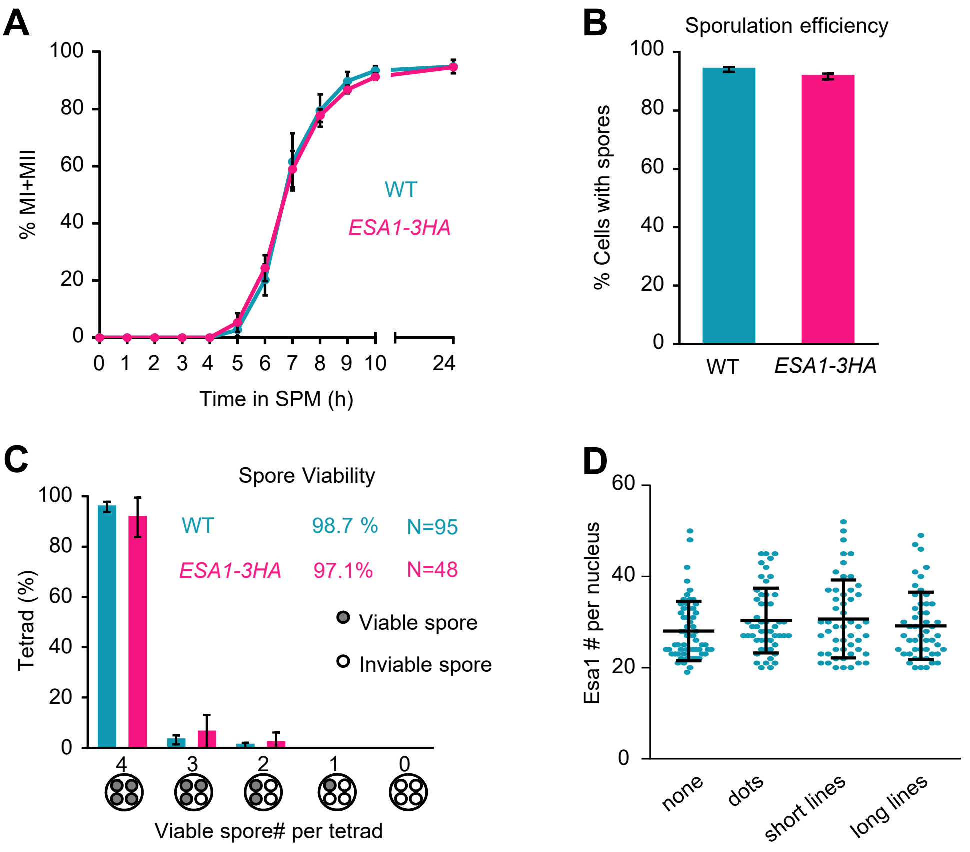

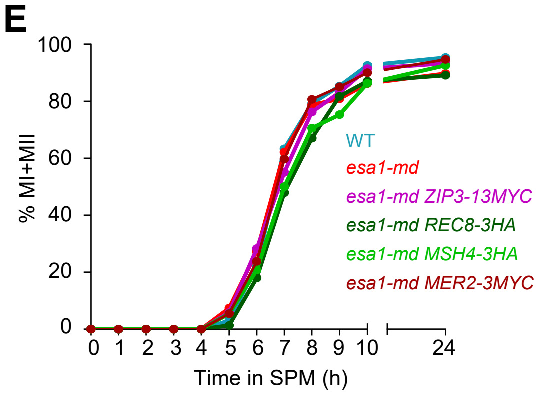

F

Strains	Sporulation frequency (%)	Spore viability (%)
WT	94.3	98.7 (N=95)
esa1-md	23.1	65.9 (N=96)
esa1-md ZIP3-13MYC	29.1	68.2 (N=96)
esa1-md REC8-3HA	28.7	72.9 (N=72)
esa1-md MSH4-3HA	20.9	72.4 (N=48)
esa1-md MER2-3MYC	18.2	73.9 (N=48)

Figure S1. Tagged proteins function normally in meiosis.

(**A**) The tagged *ESA1-3HA* strain showed normal time course. Experiments were repeated 4 times (WT) or twice (*ESA1-3HA*), and >200 nuclei were examined at each time point in each experiment. (**B**) Similar sporulation efficiency in *ESA1-3HA* and WT strains. Experiments were repeated twice for both WT and *ESA1-3HA*; > 200 cells examined in each experiment at 24h in SPM; error bar, SE. (**C**) Similar spore viability in *ESA1-3HA* and WT strains. Totally 95 (WT) and 48 (*ESA1-3HA*) tetrads were assayed. Error bar, 95% confidence interval. (**D**) Quantification of Esa1 foci from Figure 1B in different classes of meiotic nuclei. Sample size, n= 61, 52, 51 and 51 nuclei, respectively. Error bar, SD. (**E**) Time course. >150 nuclei were examined at each time point in each experiment. (**F**) Sporulation efficiency and spore viability. Sporulation efficiency was examined in > 200 cells for each strain. For spore viability, the numbers of tetrads examined were shown in the parenthesis. Samples were collected at 24h in SPM.

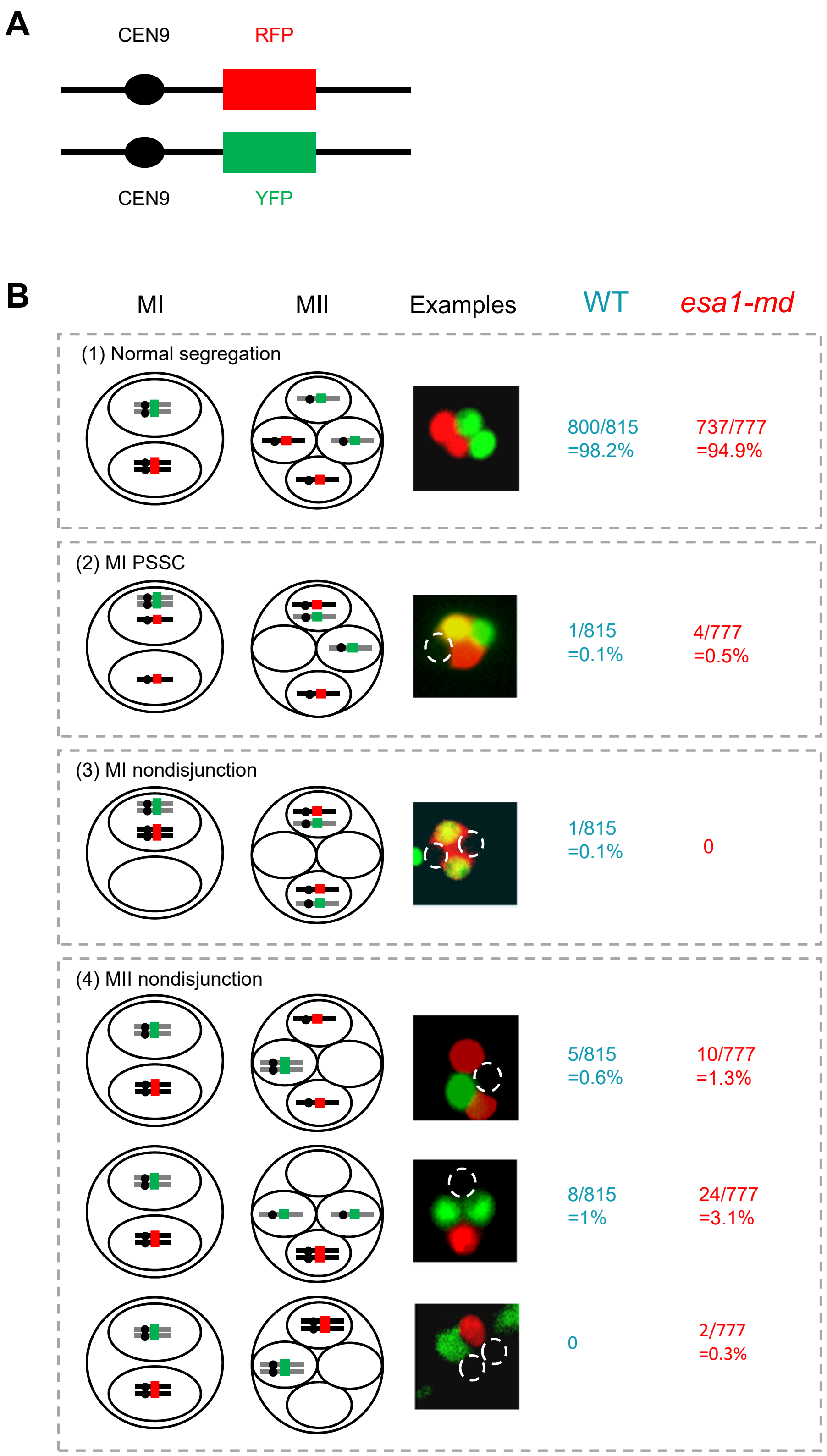

Figure S2. Increased frequency of chromosome mis-segregation in *esa1-md* strain.

(**A**) Strain configuration used for spore-specific fluorescence assay of chromosome mis-segregation. (**B**) Cartoons to show patterns of chromosome 9 segregation at meiosis I (MI) and meiosis II (MII). Corresponding examples and sample sizes examined also shown. Samples were collected at about 48h in SPM.

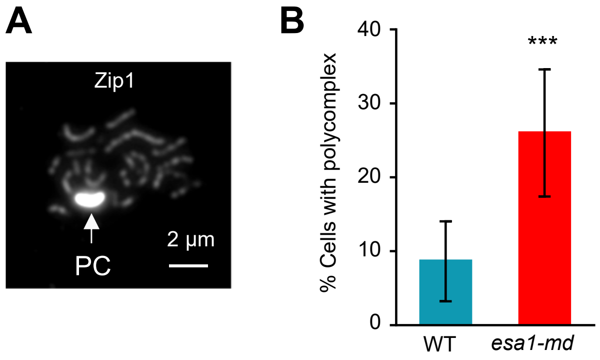

Figure S3. Increased frequency of nuclei with polycomplex in *esa1-md* strain.

(**A**) Polycomplex (PC, arrow). Samples from synchronized cultures at 5h in SPM when pachytene nuclei are most rich during the time course. Scale bar, 2 μm. (**B**) Quantification to show increased frequency of nuclei with polycomplex in *esa1-md*. N = 104 (WT) and 100 (*esa1-md*) nuclei. Error bar, 95% confidence interval; ***, p<0.001 (Two-proportion Z-test).

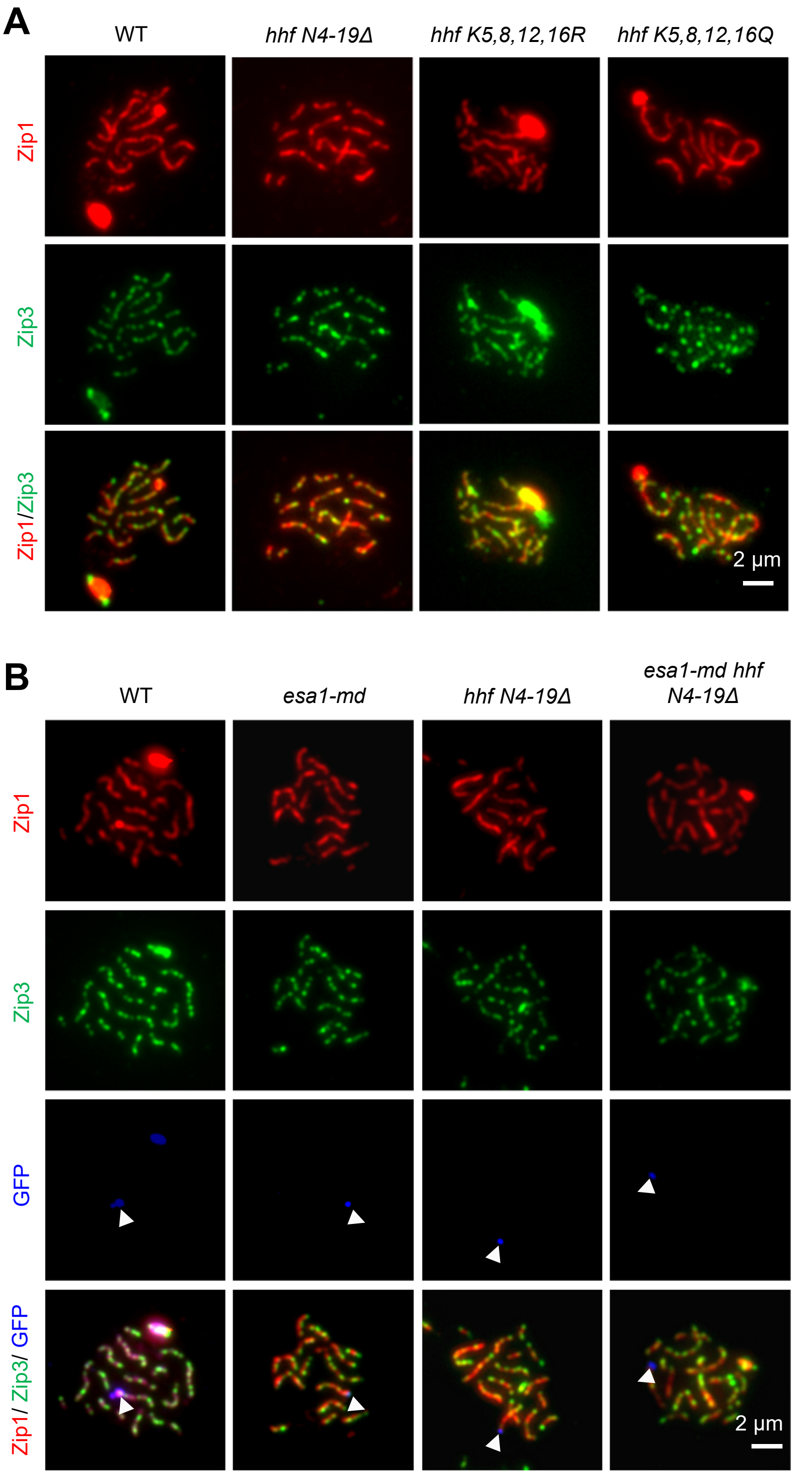

Figure S4. Visualization of Zip3 foci.

(**A**) Representative images to show Zip3 foci (green) in pachytene nuclei (judged by Zip1 staining, red). (**B**) Visualization of Zip3 foci (green) on chromosome XV (marked by the LacO/LacI-GFP spot, blue) in pachytene nuclei (Zip1 staining, red). Scale bar, 2 μm.

A

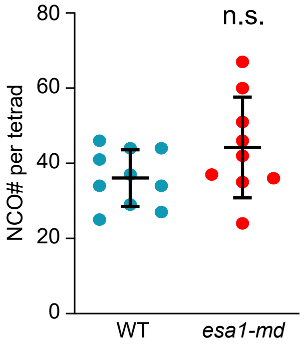

B

Chromatid interference

	WT (2:3:4 chromatids)	*esa1-md* (2:3:4 chromatids)
Observed	220:383:203	174:304:170
Expected	201.5:403:201.5	162:324:162
P value (Chi square)	0.853	0.533

Figure S5. Analyses of recombination by whole-genome DNA re-sequencing.

(**A**) The number of NCOs detected. The whole-genome DNA re-sequencing showed increased number of NCOs in *esa1-md*. This result seems contrary to decreased axis length, DSBs, and COs. However, this result probably not reflect the real situation. (1) The *esa1-md* mutant has a very low sporulation efficiency, and among them, only a small fraction of tetrads produces 4-viable spores which can be used for this analysis. Therefore, only this specific type of “meiotic nuclei” are “selected” for this mutant, which probably have different recombination behavior. (2) The *esa1-md* mutant has synapsis defect, which would promote extra DSB formation (117-118). These extra DSBs could be preferentially repaired as NCOs, which explains increased NCOs in many synapsis-defective mutants (13). (3) The heterologous strains used for this analysis may affect recombination property. (4) At least two of the nine tetrads analyzed in *esa1-md* show very high numbers of NCOs. This indicates that these 4-viable tetrads (at least the two with lots of NCOs) experienced different NCO process. N= 10 (WT) and 9 (*esa1-md*) tetrads. n.s., not significant ($p>0.05$); Student's *t* test. (**B**) Chromatid interference in WT and *esa1-md*. Chromatid interference means a CO between two nonsister chromatids affects the usage of any of these two chromatids in its adjacent COs, and this can be examined by the usage of the ratio of two-, three-, and four-strand double COs. The Chi square test shows no significant difference between the observed and expected (based on the assumption of no chromatid interference) ratios of two, three- and four-strand COs.

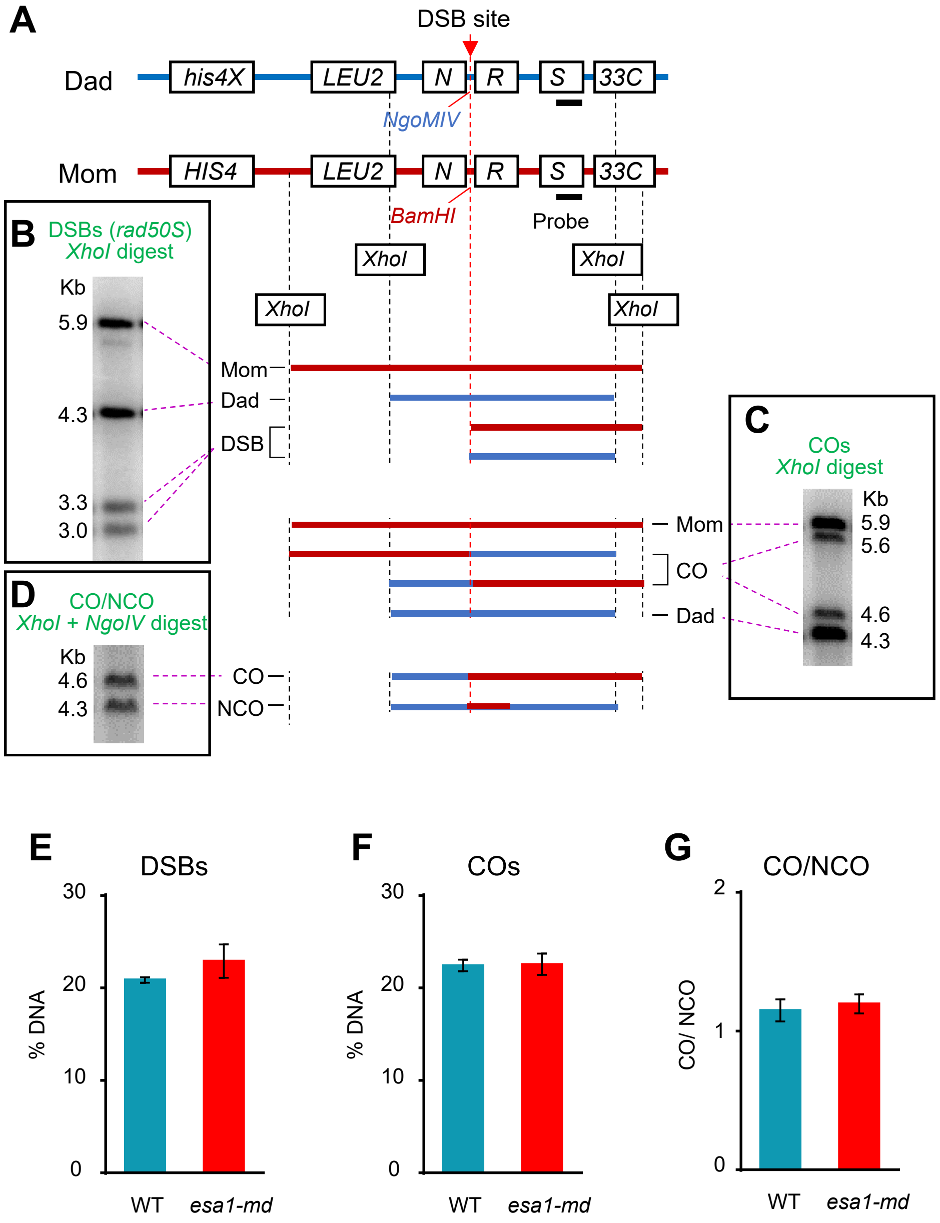

Figure S6. ESA1 depletion does not alter CO patterning at *HISLEU2* locus.

(**A-D**) The diagram of the *HIS4LEU2* hotspot and the physical assay of DSBs and recombination products. (**E-G**) Quantification of DSBs (E), COs (F), and CO/NCO ratio at *HIS4LEU2* hotspot from WT and *esa1-md* at 10h in SPM. DSBs were examined in a *rad50s* background, which blocks DSB end resection and thus accumulates DSBs to avoid possible turnover difference in WT and *esa1-md*. Error bars represent SEM from at least three independent experiments (WT) or the range from two biological replicates (*esa1-md*).

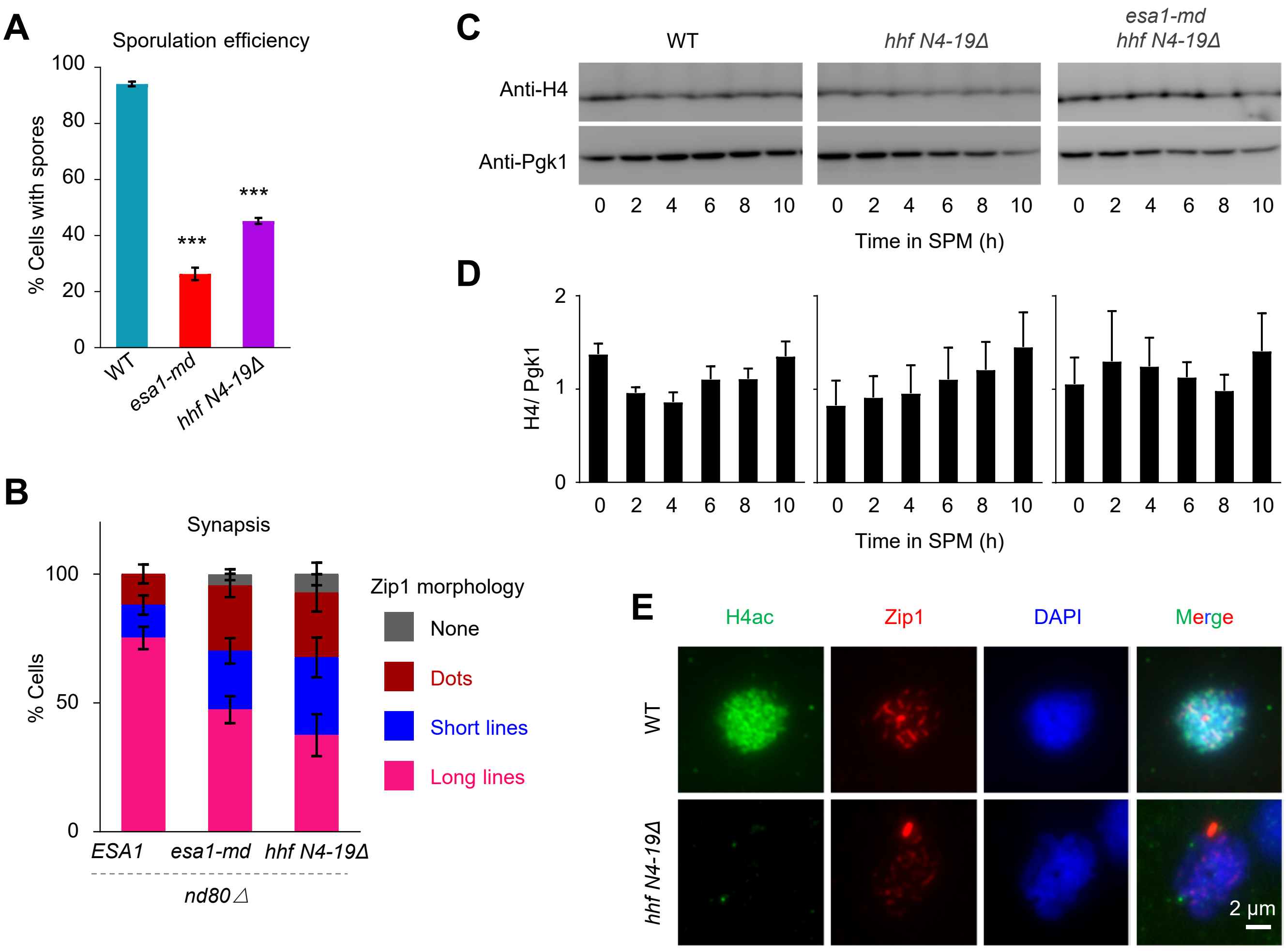

Figure S7. ***hhf N4-19Δ* showed similar defects in synapsis and sporulation as the *esa1-md.***
(**A**) Decreased sporulation efficiency in *hhf N4-19Δ* and *esa1-md* strain. Experiments were repeated twice for *hhf N4-19Δ* mutant; > 200 cells examined in each experiment. Data for WT and *esa1-md* from Figure 2C. Error bar, SE; ***, p<0.001 (Two Proportion Z-test). (**B**) Synapsis was partially impaired in the *hhf N4-19Δ* mutant. Quantification of the frequencies of nuclei with different Zip1 morphologies as in Figure 2GH. N = 136 nuclei (*hhf N4-19Δ*), and data for WT and *esa1-md* from Figure 2GH. Error bar, 95% confidence interval. (**C**) Detection of histone H4 abundance in WT, *hhf N4-19Δ*, and *esa1-md hhf N4-19Δ* by Western blot. (**D**) Quantification of (C) to show *hhf N4-19Δ* does not impair histone H4 abundance. Error bar, SE from three experiments. (**E**) Representative images of Zip1 and Histone H4 (acetyl K5+K8+K12+K16) staining in WT and *hhf N4-19Δ* strains. Samples were collected at 3-5 hours in SPM. Scale bar, 2 μm.

Supplementary Table S1. Primers for plasmid construction.

Primer#	Sequence
ZP503F	AGTCTCGAGTTTCGTTATCTTCCACGC
ZP504R	GACACTAGTTATTTCTCCTAAACCCGC
ZP507F	AGTCTCGAGCCTTAGGGCGTGCCAATAG
ZP508R	GACACTAGT TTCTTTTATTGAGACTTATT
ZP503R	TGGATGTTATCTCTTAGAATCTTTCT ACCGGACATATTATACTATATTATATTTGT
ZP504F	ACAAATATAATATAGTATAATATGTCCGGT AGAAAGATTCTAAGAGATAACATCCA
ZP1003	CTGTGCTCCACCTTGACCTAGACCTTGACCACCTTGACCTCTACCGGACATATTAT
ZP1004	CAAGGTGGTCAAGGTCTAGGTCAAGGTGGAGCACAGCGTCACAGAAAGATTCTAAGAG
ZP1005	CCTTGCTCCACCTCTACCTAGACCTCTACCACCTCTACCTCTACCGGACATATTAT
ZP1006	AGAGGTGGTAGAGGTCTAGGTAGAGGTGGAGCAAGGCGTCACAGAAAGATTCTAAGAG
ZP507R	ATGTTATCTCTTAGAATCTTTCT ACCGGACATTATTTTATTGTATTGATT
ZP508F	ATAAAATAATGTCCGGT AGAAAGATTCTAAGAGATAACATCCAAG
ZP1007	CTGTGCTCCACCTTGTCCTAGACCTTGACCACCTTGACCTCTACCGGACATTATTTTATTG
ZP1008	CAAGGTGGTCAAGGTCTAGGACAAGGTGGAGCACAGCGTCACAGAAAGATTCTAAGAGAT
ZP1009	CCTTGCTCCACCTCTTCCTAGACCTCTACCACCTCTACCTCTACCGGACATTATTTTATTG
ZP1010	AGAGGTGGTAGAGGTCTAGGAAGAGGTGGAGCAAGGCGTCACAGAAAGATTCTAAGAGAT

Supplementary Table S2. Strains used in this study.

Strain	Genotype
LZY819	*MATa/MATα, ho::hisG/", ura3/", leu2/", ZIP3-13MYC::Hygromycin B/", URA3::CYC1p-LacI-GFP/", SCP1::LacO-LEU2/"*
LZY1663	as LZY819, except *pCLB2-ESA1::KanMX4/"*
LZY3027	as LZY819, except *ESA1-3HA::KanMX/"*
LZY4656	as LZY3027, except *REC8-18MYC::KanMX4/"*
SWY89	*MATa/MATα, ho::hisG/", his3/", leu2/",CAN/can, CYH2/cyh2, CEN9:: LEU2-* $P^{YKL050c}$*RFP/CEN9::HIS3-*$P^{YKL050c}$*YFP*
LZY2953	as SWY89, except *pCLB2-ESA1::KanMX/"*
LZY3011	*MATa/MATα, ho::hisG/", ura3/", leu2/", REC8-3HA::URA3/", ZIP3-13MYC::Hygromycin B/", ndt80::LEU2/"*
LZY3445	as LZY3011, except *pCLB2-ESA1::KanMX4/"*
LZY3450	*MATa/MATα, ho::hisG/", lys2/", ura3/", leu2::hisG/", pCLB2-ESA1::KanMX/"*
LZY629	*MATa/MATα, ho::hisG/", ura3/", leu2/", HIS4::LEU2-(BamHI+ori)/ his4-x::LEU2-(NgoMIV+ori)-URA3, nuc1::hygroB/"*
LZY3032	as LZY629, except *pCLB2-ESA1::KanMX/"*
LZY4166	as LZY629, except *rad50KI81::URA3/"*
LZY3088	as LZY4166, except *pCLB2-ESA1::KanMX/"*
LZY2965	*MATa/MATα, ho::hisG/", leu2/", hhf1△N4-19/", hhf2△N4-19/", ZIP3-13MYC::Hygromycin B/", leu2::LacI-GFP::KanMX/", Scp1::LacO::LEU2/"*
LZY4657	as LZY2965, except *pCLB2-ESA1::NAT/"*
LZY2700	(S96) *MATa, ho::hisG, lys5*
LZY2716	as LZY2700, except *pCLB2-ESA1::KanMX*
LZY2701	(YJM789) *MATα, lys2, cyh*
LZY2713	as LZY2701, except *pCLB2-ESA1::KanMX*
LZY4047	*MATa/MATα, ho::hisG/", leu2/", hhf1 K5,8,12,16R/", hhf2 K5,8,12,16R/", ZIP3-13MYC::Hygromycin B/", leu2::LacI-GFP::KanMX/", Scp1::LacO::LEU2/"*
LZY4774	*MATa/MATα, ho::hisG/", leu2/", hhf1 K5,8,12,16Q/", hhf2 K5,8,12,16Q/", ZIP3-13MYC::Hygromycin B/+*
LZY4775	*MATa/MATα, ho::hisG/", ura3/", leu2/", lys2/", MER2-3MYC::NAT/"*
LZY4776	as LZY4775, except *pCLB2-ESA1::KanMX/"*
LZY4777	*MATa/MATα, ho::hisG/", ura3/", leu2/", lys2/", mnd1::NAT/"*
LZY4778	as LZY4777, except *pCLB2-ESA1::KanMX/"*
LZY4545	*MATa/MATα, ho::hisG/", ura3/", MSH4-6HA::NAT/"*
LZY4779	as LZY4545, except *pCLB2-ESA1::KanMX/"*
LZY4780	*MATa/MATα, ho::hisG/", ura3/", leu2/", leu2::LacI-GFP::KanMX/", WT/tel4::226xLacO::NAT, WT/trp1::226xLacO::NAT, REC8-3HA::URA3/", ndt80::LEU2/"*
LZY4781	as LZY4781, except *pCLB2-ESA1::KanMX/"*
LZY4782	*MATa/MATα, ho::hisG/", ura3/", REC8-3HA::URA3/", ZIP3-13MYC::Hygromycin B/"*
LZY4783	as LZY4782, except *pCLB2-ESA1::NAT/"*

LZY3453	*MATa/MATα, ho::hisG/", leu2/", lys2/", Esa1-3HA::KanMX/"*
LZY4821	*MATa/MATα, ho::hisG/", ura3/", leu2/", REC8-3HA::URA3/", pCLB2-ESA1::KanMX/", ndt80::LEU2/"*
LZY3017	as LZY3011, except *pCLB2-PDS5::KanMX/"*
LZY4822	as LZY3017, except *pCLB2-ESA1::NAT/"*

Supplementary Table S3. Primers for RT-qPCR.

Primer#	Sequence
P SPO71 F	GCGATGCTTTACGCTTTGGT
P SPO71 R	CTTGCCGCTCACACCTAACT
P SPO20 F	ACGTAGCCGACCGAAAGATT
P SPO20 R	ATACGCTGTCTTTCCACCCC
P SPO73 F	GCAGGCAGTAGCAGTTCACA
P SPO73 R	AGACCCTTCTTCTCACCAAGC
P SPR1 F	TGGGGGAAAGATGCCGTAAG
P SPR1 R	TGTTTTCTCTCTTCGGGCCA
P SPC42 F	AAGAACCCTCTCGGTCCTCA
P SPC42 R	CGCTTCAGCTCTGCACTTTC
P PDS1 F	ACAAGGAGGGAAGGAGGTGT
P PDS1 R	TGGTTGGCCCTACTACGACT
P CLB1 F	TCCCAAGGACCATTCTCGGT
P CLB1 R	TGCTGCTTTTCTTGCTCGGA
P CLB4 F	TTTCGGTCGGTTCCCAATCC
P CLB4 R	TGAACGGCCATGTAAGCTCT
P ACT1 F	AGGAAATCACCGCTTTGGCT
P ACT1 R	TGTGGTGAACGATAGATGGACC

Supplementary Table S4. Genes with altered transcription abundances from RNA-seq experiments in *esa1-md* compared with WT.
